# Male scent-marks predict fitness via socio-spatial dominance, but not female choice, in a lacertid lizard

**DOI:** 10.1098/rspb.2026.0863

**Published:** 2026-11-04

**Authors:** Javier Abalos, Alicia Bartolomé, Guillem Pérez i de Lanuza, Fabien Aubret, Tobias Uller, Pau Carazo, Enrique Font, Roberto García-Roa

**Affiliations:** ^1^ Ethology, Instituto Cavanilles de Biodiversidad y Biología Evolutiva, University of Valencia, Valencia, Spain; ^2^ School of Natural Sciences, University of Tasmania, Hobart, Australia; ^3^ CNRS, Station d'Ecologie Théorique et Expérimentale, Moulis, Ariège, France; ^4^ Department of Biology, Lund University, Lund, Sweden

**Keywords:** animal communication, pheromones, chemical signals, reptiles, sexual selection, mate choice, male–male competition, territoriality, lizard

## Abstract

Chemical communication via scent-marks is widely recognized as a key mediator of sexual selection in lizards, yet it remains poorly understood how scent-mark composition ultimately impacts male fitness in nature. Male scent-marks are often proposed to function as condition-dependent honest sexual signals mediating female choice, but an alternative hypothesis is that they primarily function and have evolved in the context of male–male competition. We provide a comprehensive test of these competing hypotheses. We studied common wall lizards (*Podarcis muralis*) in outdoor enclosures and combined chemical analyses of scent-marks with detailed behavioural and spatial data, genetic parentage assignment and path analysis to quantify how scent-mark composition relates to mating behaviour, contest outcomes, sperm competition and male reproductive success. We found no evidence that females assess mates based on candidate compounds proposed as signals of male quality. Instead, female settlement and fertilization patterns were driven by resource distribution and spatial proximity. Furthermore, several compounds correlated with dominance, and male–male competition emerged as the primary driver of reproductive success, strongly suggesting that scent-marks mediate territorial dynamics, rather than female choice.

## 1. Introduction

Sexual selection is a major evolutionary force shaping phenotypic diversity, and chemical communication is one of the main avenues through which it operates [1,2]. Lizards exhibit a striking diversity in sexually selected traits, with social interactions mediated through multiple signalling modalities, including visual displays, coloration and chemical communication [3–5]. Because these modalities differ in how information is transmitted (e.g. chemical signals persist without the emitter), they are likely to be shaped by different selective pressures and provide complementary sources of social information [6]. Males of many lizard species deposit scent-marks onto substrates, consisting of serous secretions produced by epidermal glands located in the precloacal or femoral region [7]. While scent-mark composition varies between individuals and species, lipids are present in the scent-marks of all lizard families studied [5,8], and some have been proposed to act as honest signals of male quality mediating sexual selection through both male–male competition and female mate choice [7,9,10]. Chemical cues and scent marking participate in mating and competitive interactions across lizard families [11–13], yet studies connecting scent-mark composition to sexual selection are unusually concentrated in lacertids [10,14]. This literature provides substantial evidence that scent-marks mediate the assessment and/or discrimination of different conspecific socio-sexual characteristics, but the relative importance of female mate choice versus male–male competition in the evolution and function of scent-marks remains contested [9,15].

Considerable attention has focused on the hypothesis that female lacertid lizards assess scent-mark composition to obtain information about individual quality [16–18]. Several compounds have been identified as potential quality signals, including α-tocopherol (vitamin E), oleic acid and two vitamin D precursors (i.e. ergosterol or provitamin D_2_, and cholesta-5,7-dien-3-ol or provitamin D_3_). Because these compounds play essential roles in physiology and development, allocating them to scent-marks may entail trade-offs [10], which could ensure honesty via condition-dependent signalling [19]. For example, some studies have reported female preferences for male scent-marks with high levels of vitamin E [20,21], while carboxylic acids (hexadecanoic and octadecanoic acid) and steroids (campesterol, sitosterol and ergosta-5,8-dien-3-ol) have been linked to stress physiology [22]. Altogether, the interplay between physiological trade-offs and female responses has provided a rationale for a role of scent-mark composition in female mate assessment (reviewed in [10,14]). However, most evidence linking variation in specific compounds to female attraction is indirect and largely based on laboratory assays. Whether such preferences translate into mate choice and ultimately reproductive success under ecologically realistic conditions remains largely untested.

The socio-spatial organization of many lacertids provides further reason to question whether female mate assessment is the primary pathway linking scent-mark composition to male reproductive success [3,9,23–25]. First, most lacertid lizards show a resource-based territorial system, which provides little opportunity for female choice. Females typically occupy smaller home ranges than males [26,27], making mate searching costly in terms of time, energy and predation risk [26,28]. Moreover, increased exposure to males results in male harassment, with significant costs to females [29,30]. Second, females settling in conspecific high-quality territories should, even in the absence of information about male quality, indirectly mate with males of high resource-holding potential [31,32]. Consistent with this view, while ample evidence shows that lacertid lizards use scent-marks for species, sex and mate recognition [24,33], whether females employ scent-marks for mate assessment remains contested [3,9].

Alongside its role in intersexual interactions, there are good reasons to expect that scent-mark composition may play an important role in male–male competition among lacertids [9,34,35]. Mounting evidence indicates that males are the sex more reliant on chemosensory processing in social and reproductive contexts [3,25]. Male lacertid lizards have larger olfactory bulbs and higher rates of seasonal adult neurogenesis in chemosensory brain regions than females [9,36]. Behavioural studies show that males assess scent-marks based on both pheromones and individually variable signature mixtures [2], enabling them to evaluate the competitive ability of unfamiliar rivals [37,38] and to recognize familiar individuals based solely on scent-marks [25]. Variation in scent-mark composition arising from ontogeny, parasites, diet or basking may provide information relevant to male–male assessment [34,39]. However, although scent-mark composition can influence male–male contest outcomes [40], whether these effects translate into differences in male reproductive success remains largely unknown.

To address these gaps in knowledge, we examined how variation in male scent-mark composition relates to socio-sexual behaviour and reproductive success in the European common wall lizard (*Podarcis muralis*), a widely used species in studies of lizard chemical communication [9,41]. We focused on the main candidate compounds previously suggested to contribute as signals of individual quality in the context of female choice: cholesterol, α-tocopherol, ergosterol (provitamin D_2_), oleic acid, hexadecanoic acid, octadecanoic acid, campesterol, cholesta-5,7-dien-3-ol (provitamin D_3_), sitosterol and ergosta-5,8-dien-3-ol [10,14,42]. We conducted the study in outdoor mesocosms replicating natural vegetation, weather conditions, prey availability, light cycles and habitat heterogeneity, allowing spontaneous social interactions and precise estimates of male reproductive success.

## 2. Materials and methods

### (a) Study species, sampling and morphological measurements

To assess the link between scent-mark composition and sexual selection, we studied the European common wall lizard (*P. muralis*), which produces scent-marks from femoral pores on the ventral thighs (figure 1a). We captured 180 lizards (90 females and 90 males) by noosing from 20 capture localities spanning 12 geographic areas in the Cerdanya Plateau (Eastern Pyrenees; supplementary material, table S1). Following previous studies, we restricted sampling to adults (snout-to-vent length, SVL ≥ 56 mm) [27,43]. Males were captured immediately before the onset of the experiment (May 2018). Females were captured at the end of the previous breeding season (September 2017) to avoid gravidity and housed under semi-natural outdoor conditions until the experiment [27] (see supplementary material, appendix S1 for more information on female husbandry).

**Figure 1.**
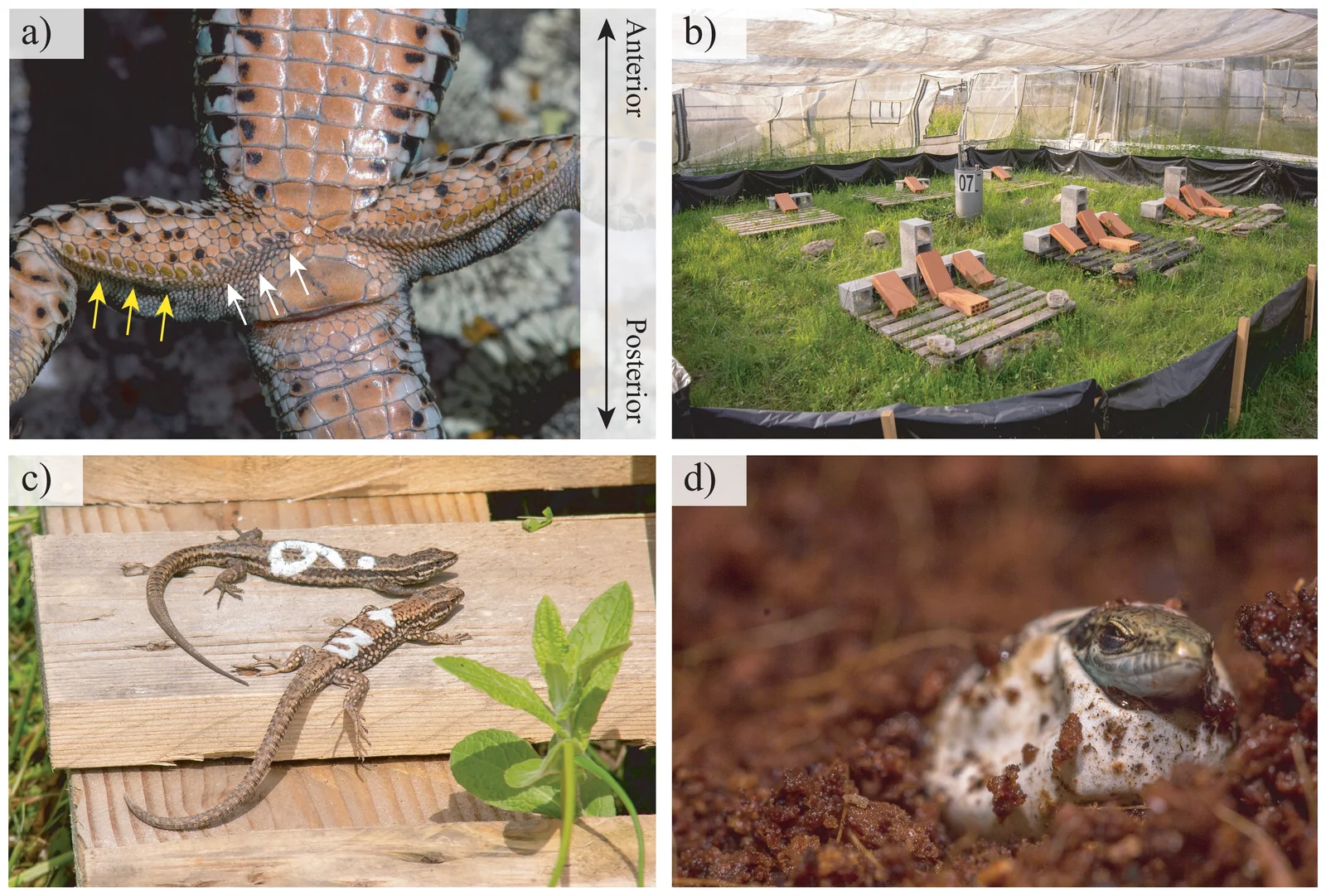
(a) Detailed view of the pelvic region of an adult male *Podarcis muralis*, showing the femoral pore rows along the ventral surface of each hindlimb. A waxy secretion can be observed in the femoral pores indicated with yellow arrows and is absent in pores indicated with white arrows. (b) A view of one of the experimental enclosures used for the mesocosm experiment at the Metatron research facility (CNRS; Caumont, France). (c) Male (marked with the number ‘3’) and female (marked with the number ‘6’) pair of *P. muralis* lizards engaged in co-perching behaviour within an experimental enclosure. (d) Lizard hatching from one of the eggs resulting from clutches incubated in the laboratory. Parentage analyses of these offspring enabled us to reconstruct mating patterns, estimate sperm competition intensity and quantify reproductive success, thereby identifying the behavioural and chemical traits that shaped male fitness within the enclosures.

Two days before the onset of the experiment, we measured the SVL (± 0.1 mm) and mass (± 0.01 g) of each lizard with a ruler and a spring balance (Pesola), as well as head length (HL) in males using a digital caliper (± 0.01 mm; Mitutoyo, Telford, UK). We estimated body condition (BCI) for each sex as residuals from a log–log regression of body mass on SVL. The relationship was linear, and residuals were homoscedastic [44,45]. Likewise, in males we estimated an index of relative head size (HL res) by obtaining the residual from a least-squares linear regression of log(HL) against log(SVL). We also removed approximately 5 mm from the tail tip of each individual and preserved the tissue in 90% ethanol for genetic analyses.

### (b) Scent-mark composition analyses

Male femoral gland secretions (i.e. considered the main contributors to scent-marks; [3,46]) were collected from the left hindlimb of each male and analysed using gas chromatography–mass spectrometry (GC-MS). We first obtained a semi-quantitative overview of scent-mark composition using scan-mode analyses. Compounds were identified using authentic-standard retention times and NIST 2017 mass-spectral matches [21,47]. Because scan-mode analyses provide only a broad compositional overview, we subsequently targeted ten compounds allegedly implicated in sexual selection [10,14]. Samples were treated with hexane containing n-docosane D46 as an internal standard (IS), adjusted to secretion mass, and compound abundances were expressed relative to the IS. This allowed compounds to be quantified independently of one another [48] and compared among individuals. Relative abundances were extracted from diagnostic ions and retention times.

Reliable targeted data were obtained for 78 of 90 males. Samples showing unreliable chromatographic signals despite repeated injections were excluded. Because secretion replenishment time is unknown, we sampled only one hindlimb to avoid depleting males of their entire scent-mark reservoir. Full analytical procedures, identification criteria, comparisons between scan and targeted analyses, and analyses of geographic variation are provided in supplementary material, appendix S1 (table S2; figure S1).

### (c) Preprocessing of scent-mark composition data

Compound abundances were strongly right-skewed, contained occasional extreme values, differed substantially in scale and showed considerable covariation (supplementary material, figures S2–S3). To limit the influence of extreme observations without excluding individuals, abundances were Winsorized at the 95th percentile. Zeros were replaced with compound-specific pseudo-counts equal to 10% of the smallest non-zero abundance [49], after which abundances were log-transformed and z-standardized. To reduce multicollinearity, we retained only compounds with pairwise correlations less than or equal to |0.6| [50], resulting in seven variables: cholesta-5,7-dien-3-ol, ergosterol, octadecanoic acid, campesterol, oleic acid, α-tocopherol and sitosterol (supplementary material, table S3). As a sensitivity analysis, we also calculated Spearman correlations between untransformed compound abundances and key measures of male competitive, socio-spatial and reproductive performance (supplementary material, figure S4). Full preprocessing details and rationale are provided in supplementary material, appendix S1.

### (d) Mesocosm experiment

We studied social behaviour in *P. muralis* using 10 semi-natural outdoor enclosures (47 m² each) located at the Metatron facility, an annex to the CNRS Station d'Écologie Théorique et Expérimentale (SETE) in Moulis (Caumont, France). Within each of these enclosures, we created two types of sites that varied in structural complexity, which is known to influence habitat use and social dynamics in lizards [51]. Following a previous semi-natural experiment in this species [52], we used a relatively high lizard density and fewer structured sites than males to promote competition for spatial and thermoregulatory resources. Each site consisted of a wooden pallet (approx. 1.2 m^2^) with differing numbers of bricks, cinderblocks, rocks and logs piled above, which acted both as shelter and perching/basking sites. We arranged three high-quality (HQ) and three low-quality (LQ) sites in two rows along the north–south axis, separated by a line of six rocks, which we also considered low-quality sites (figure 1b).

On 23 May 2018, nine males were released into each enclosure and monitored for 7 days before nine females were added. Lizards fed primarily on naturally occurring arthropods, supplemented with live mealworms and crickets evenly distributed across sites of different quality. To allow for individual identification, lizards were both permanently marked on their ventral scales using a disposable cautery unit [53] and painted on their dorsum with a white Edding©751 marker (figure 1c) [54]. To minimize prior experience effects, males and females assigned to each enclosure were deliberately mixed among capture localities, with all individuals originating from sites separated by at least 300 m. Individuals were also assigned to minimize variation in SVL within each sex. Males were removed after one month and released at their capture sites, while females were housed individually until oviposition [55]. We lost 22 clutches owing to failure to lay or unrecovered eggs within enclosures. For the remaining 68 females, we counted the number of fertile and infertile eggs within each clutch. Of the 385 eggs produced, 60% were fertile (*N* = 230), and 90% of those fertile eggs produced viable hatchlings (*N =* 208). The relatively high rate of infertility may have resulted from the delay between females emerging from hibernation and their introduction to males (supplementary material, appendix S1), as some females may have been too advanced in their reproductive cycle for successful mating and fertilization. Importantly, however, infertility rates did not differ between females occupying high- and low-quality habitats (beta-binomial generalized linear mixed model (GLMM) on fertilization success: *Z =* 1.24, *p =* 0.215). Fertile eggs were incubated, and upon hatching (figure 1d), each of the born juveniles was measured (SVL; ± 0.1 mm), weighed (± 0.001 g), sampled for DNA, permanently marked and released into the outdoor tanks in SETE Moulis [4,27].

### (e) Behavioural observations

Spatial and social behaviour was recorded daily between 9.00 and 19.30 from 23 May to 22 June. Positional data were collected using scan sampling at 2.5-hour intervals, while social interactions were recorded using behaviour sampling during standardized observation sessions (supplementary material, table S4). To delimit distinct behavioural interaction bouts, consecutive encounters between the same individuals were considered separate when participants remained more than 30 cm apart for at least 2 min. Full observational procedures are detailed in appendix S1 in the supplementary material.

### (f) Behavioural analyses

#### (i) Spatial behaviour

Habitat use was quantified using fixed-kernel density estimates [56]. Each lizard was assigned residency at a specific site based on the location of peak density within the 50% kernel estimate (see supplementary material, appendix S1 for more details). Across the experiment, we recorded 7189 resightings from 614 scans. Females had a significantly higher probability of being found in high-quality sites (probability = 0.75 [CI_95_: 0.64–0.83]) than males (0.22 [CI_95_: 0.14–0.32]), with an odds ratio of 10.7 (Tukey-adjusted *p* < 0.001) (supplementary material, table S5; [4,27]).

#### (ii) Social behaviour

We categorized interactions as intrasexual (competitive and non-competitive) or intersexual (reproductive and non-reproductive). Intrasexual interactions were considered competitive when one lizard (the loser) employed fast-paced locomotion to flee from another lizard (the winner), showing raised-body display behaviour and/or physical aggression (i.e. bite or chase) (supplementary material, table S4). Male–male contests were analysed using Bradley–Terry models fitted to the observed matrix of male–male contest outcomes within each enclosure [57]. Baseline models without predictors were used to derive dominance ranks (range: 1–9), and extended models incorporating individual and contest-level predictors were used to examine determinants of contest outcomes (see below).

### (g) Parentage analysis

Parentage was assigned using multilocus microsatellite genotypes and likelihood-based methods [27]. We could reliably assign paternity to every offspring examined (strict: 208 juveniles, relaxed: 230 juveniles). Detailed methods for parentage analysis are provided in appendix S1 in the supplementary material. We quantified individual fitness as: mating success (i.e. the overall number of different mates with whom a lizard conceived offspring) and reproductive success (i.e. the total number of viable hatchlings sired). Since selection depends on relative rather than absolute fitness, we then divided the fitness measures of each lizard by the mean for all same-sex conspecifics within its enclosure. In addition, to evaluate male differences in sperm competition intensity, for each male we determined the average number of competitors with which he shared paternity by dividing the number of co-sires by the number of mates (SCI = sperm competition intensity).

### (h) Statistical analyses

#### (i) Structural equation modelling

To test relationships among chemical compounds, socio-spatial behaviour and fitness-related variables, we applied piecewise structural equation modelling (pSEM) implemented in the R package *piecewiseSEM* [58], combining GLMMs fitted with *glmmTMB* [59] and *lme4* [60]. Piecewise SEM allows modelling systems in which variables act as both predictors and responses while accommodating mixed-effects structures and non-Gaussian error distributions [61,62].

We first examined associations among key socio-spatial and fitness variables using Spearman rank correlations to guide construction of an initial biologically informed pSEM (supplementary material, figure S5). The initial pSEM (supplementary material, figure S6) included direct effects of dominance, high-quality residency, co-perchings per female and mating success on reproductive success, as well as effects of dominance, high-quality residency and co-perchings on mating success and sperm competition intensity. Mass and HL residuals were selected because males were size-matched within enclosures and BCI was highly correlated with mass (supplementary material, figure S3). Model fit was assessed using Shipley's test of directed separation [61,63]. Based on d-separation tests, a direct effect of ergosterol on mating success was added. Because males without offspring lacked estimates of sperm competition intensity, we repeated the analysis excluding these individuals (*N =* 25) to confirm robustness of inferred pathways. Further details are in appendix S1 in the supplementary material.

#### (ii) Models on male–male competition

Building on the pSEM results, we focused on the role of chemical compounds in mediating dominance and high-quality site residency. We first tested whether scent-mark composition differed among males occupying high-quality, low-quality and rock sites using robust multivariate analysis of variance (MANOVA) [64], followed by compound-specific robust ANOVAs when covariance assumptions were violated [65. Although rock sites are classified as low quality, we treated them separately as a conservative approach; pooling them with low-quality sites yielded identical results.

To investigate the relative importance of scent-mark composition and contest-related traits in predicting contest outcomes, we fitted Bradley–Terry models (R package BradleyTerry2; [57]). Bradley–Terry models accommodate repeated participation of individuals across contests by estimating individual competitive ability from the network of pairwise outcomes. Male predictors included body mass, HL residuals and the relative abundance of seven compounds. Contest-level predictors included display behaviours (raised-body and type II foot shakes; supplementary material, table S4), residency (whether the contest occurred within the male's site) and prior contest experience, defined by whether each contestant had won its most recently observed contest [66]. Models were simplified using backward deletion and likelihood ratio tests (α = 0.05) [67]. To explore temporal dynamics, we fitted three models: a global model (*N* = 544), an early-phase model (days 1–9; *N* = 137), and a late-phase model (day 10 onward; *N* = 407). Further details are in appendix S1 in the supplementary material.

#### (iii) Models on fertilization success

To test whether females bias fertilization towards high-quality males, we modelled per-egg fertilization success using mixed-effects beta-binomial models. For each reproducing female, the response was the number of eggs fertilized by a male relative to other potential sires. Predictors included male dominance, chemical relative abundances and spatial proximity, measured either as same-site residency (binary) or parental site-quality combination (four female–male levels: high–high, low–low, high–low, low–high). We fitted two models differing only in the proximity metric. Mother and male identities were included as random effects to account for repeated measures. For males lacking chemical data, compound values were set to the population mean (*z*-score = 0) to retain the full choice set; complete-case models confirmed robustness.

#### (iv) Model fitting and selection

All models were fitted in R using mixed-effects frameworks [59,60], with predictors centred and scaled and experimental cell included as a random effect. Model assumptions and fit were evaluated using simulated residuals, and model selection was based on corrected Akaike information criterion (AICc) [68]. Piecewise structural equation models were simplified by backwards selection from a saturated model [69], and uncertainty in standardized effects was quantified using bootstrapped confidence intervals [70]. Exhaustive model selection and model averaging were used to refine inference on individual pathways [71], with significance assessed using confidence intervals and relative variable importance [67]. All analyses were conducted in R v. 4.2.2. [72]. Full details of model fitting, diagnostics, selection criteria and robustness checks are provided in appendix S1 in the supplementary material.

## 3. Results

### (a) Scent-marks and male fitness

The structure, strength and direction of the pathways in the pSEM supported an indirect effect of scent-mark composition on male relative reproductive success (figure 2). Importantly, the non-significant Fisher's *C* statistic (Fisher's *C* = 27.12, d.f. = 44, *p* = 0.979) indicates that the model adequately fits the data and that no major causal pathways appear to have been omitted (supplementary material, tables S6 and S7).

**Figure 2.**
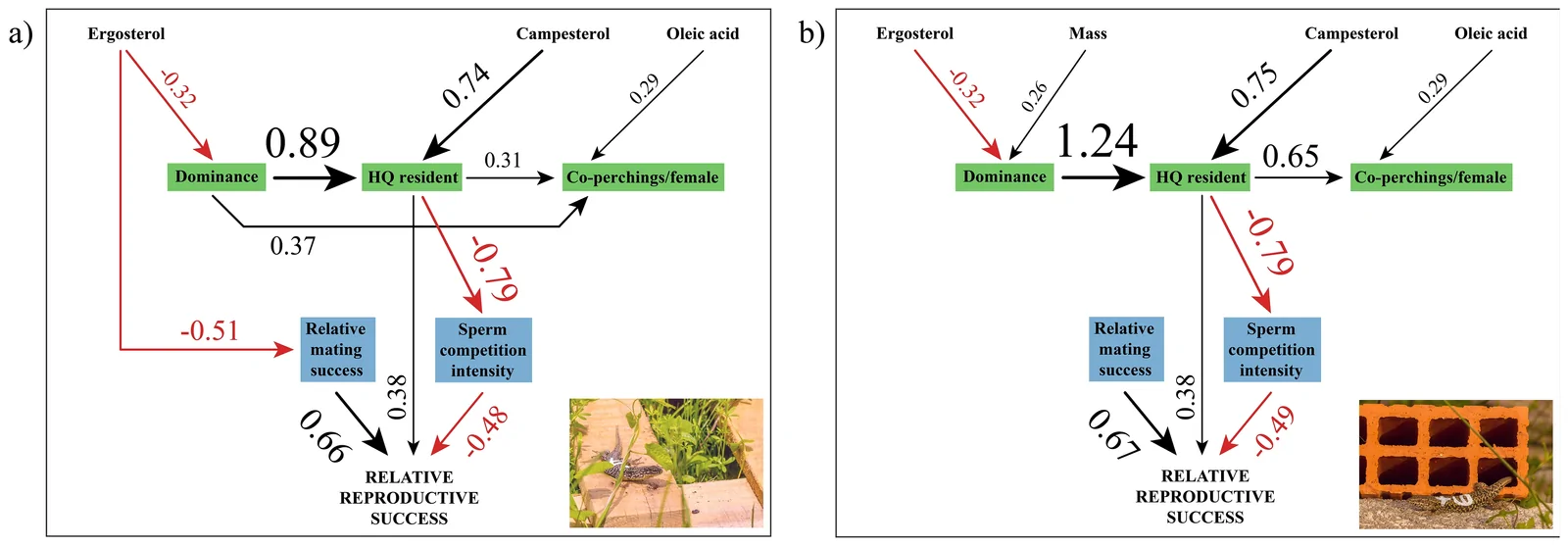
(a) Path diagrams illustrating the best-supported network of directional hypotheses from the piecewise structural equation modelling (pSEM) analysis fitted with the complete dataset, or (b) excluding the males without offspring. Arrows represent supported causal relationships among morphological predictors, scent-mark composition, socio-spatial behaviour and relative reproductive fitness. Arrow direction indicates the hypothesized causal flow; arrow thickness, font size and accompanying values reflect standardized path coefficients from the pSEM, multiplied by 10 for visualization (supplementary material, a: table S8; b: table S11). Black arrows denote positive effects, while red arrows denote negative effects. Note that standardized coefficients obtained via bootstrapping differ in scale from the corresponding estimates in tables S7 (a) and S9 (b) in the supplementary material.

Three of the seven examined compounds (i.e. ergosterol, campesterol and oleic acid) were retained in the final model (supplementary material, table S8), suggesting that they may influence key mediating traits—such as dominance and a high-quality territory—that ultimately affect male fitness. Reproductive success (i.e. the number of offspring sired, divided by enclosure mean) was found to result from the combined effect of mating success (bootstrapped standardized *β* [CI_95_] = 0.07 [0.04, 0.13]), sperm competition intensity (*β* [CI_95_] = –0.05 [–0.13, –0.00]) and a high-quality site (*β* [CI_95_] = 0.04 [0.00, 0.09]), confirming that exclusive access to multiple females is the key determinant of male fitness. Ergosterol emerged as a particularly interesting compound, as it showed a negative effect on both dominance (*β* [CI_95_] = –0.32 [–0.43, –0.17]) and mating success (*β* [CI_95_] = –0.05 [–0.07, –0.03]). Dominance had a strong effect, increasing the odds that a male occupied a high-quality site (*β* [CI_95_] = 0.09 [0.06, 0.14]), but these odds were also positively affected by campesterol levels (*β* [CI_95_] = 0.07 [0.04, 0.15]). High-quality site residency had a direct impact on reproductive success, but also an indirect one through its negative effect on sperm competition intensity (*β* [CI_95_] = –0.08 [–0.21, –0.03]). Owing to the high density of females in high-quality sites (see below), resident males were found to guard a higher number of different females (supplementary material, figure S5), while also engaging in a higher number of co-perching interactions per female (*β* [CI_95_] = 0.03 [0.02, 0.05]). Co-perching interactions also increased with dominance (*β* [CI_95_] = 0.04 [0.02, 0.06]) and levels of oleic acid (*β* [CI_95_] = 0.03 [0.02, 0.04]).

We found no evidence that dominance or residency at a high-quality site predicted mating success (i.e. the number of females sired). This suggests that, although some males obtain copulations through territory ownership and mate guarding, others probably rely on opportunistic mating. The direct effect of high-quality sites on male reproductive success, independent of mating success and sperm competition intensity, suggests that males in high-quality sites gained greater fitness from each mating (supplementary material, table S8; figure 2). Consistent with this, males in high-quality sites mated with females that were slightly larger on average (mean SVL ± SEM: high-quality: 66.9 ± 1.0 mm; low-quality: 65.6 ± 0.6 mm), although this difference was not statistically supported (linear mixed model (LMM): *t* = 1.1, *p* = 0.285). Likewise, females mated by males in high-quality sites were slightly more fecund (6.1 ± 0.4 versus 5.5 ± 0.2 fertile eggs), although this difference was also non-significant (LMM: *t* = 1.3, *p* = 0.211).

The best-fitting pSEM excluding males without offspring exhibited a similar pathway structure to the model described above, with chemical compounds exerting indirect effects on key mediators of reproductive success (Fisher's *C* = 34.52, d.f. = 50, *p* = 0.953; supplementary material, table S9; figure 2). A notable distinction from the previous model was the lack of support for a direct effect of ergosterol on mating success in the best-supported pSEM model. In addition, we found evidence of a positive effect of mass on dominance (*β* [CI_95_] = 0.03 [0.00, 0.05]), and a strong positive effect of high-quality sites on co-perching (*β* [CI_95_] = 0.07 [0.05, 0.09]) (supplementary material, tables S10 and S11).

### (b) Scent-marks and male–male contests

The Bradley–Terry model successfully predicted the contest outcome in 84% of 544 cases. Behaviours, residence and previous wins were more strongly associated with contest outcome than scent-mark composition (supplementary material, table S12). Raised-body displays markedly increased the odds of winning by a factor of 18, while type II foot shakes were linked to a threefold increase in the odds of losing. Resident males (i.e. males settled at the site where the contest took place) were three times more likely to prevail, and individuals that had won their previous contest were twice as likely to win the next one. Among targeted compounds, a 1 s.d. advantage in either cholesta-5,7-dien-3β-ol or α-tocopherol was associated with nearly a twofold increase in the odds of winning. By contrast, higher levels of ergosterol were linked to a halving of those odds. A positive effect of cholesta-5,7-dien-3β-ol and α-tocopherol was also supported by results from the dominance component model of the pSEM (supplementary material, table S7). The early-contest model (interactions from days 1–9; *N* = 137) predicted outcomes with 72% accuracy, while the late-contest model (day 10 onwards; *N* = 407) achieved 85% accuracy. Coefficients for compounds and raised-body displays were higher in early contests, whereas the effects of type II foot shakes, residency status and prior contest history became more pronounced in later stages of the experiment (supplementary material, table S12; figure 3).

**Figure 3.**
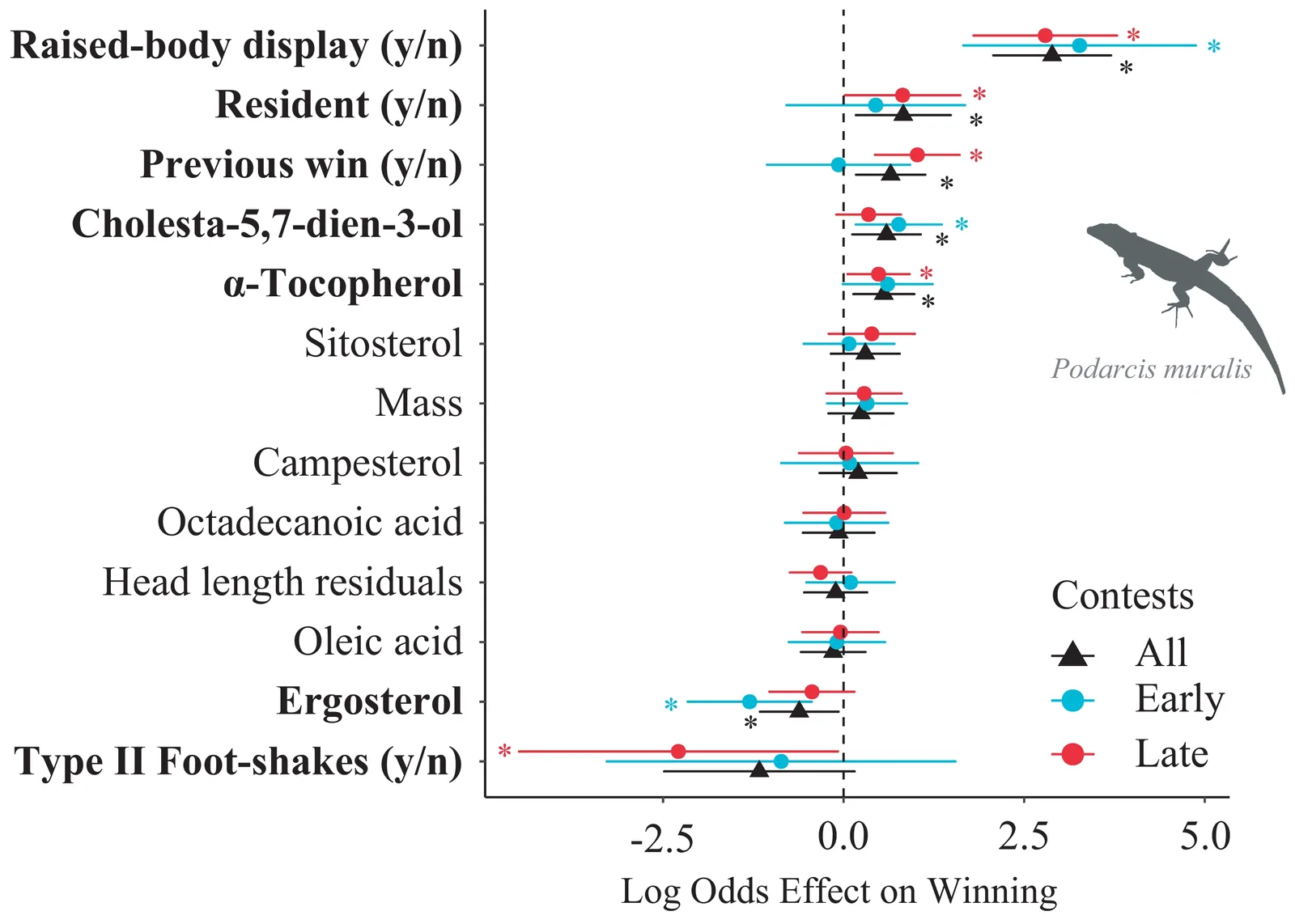
Forest plot showing model coefficients (log-scale effects on odds ratios ± CI_95_) for each of the predictors considered in three Bradley–Terry models; an all-contests model on the full dataset of male–male competitive interactions (*N* = 544), an early-contests model on the interactions from days 1–9 (*N* = 137) and a late-contests model on the interactions observed from day 10 onwards (*N* = 407). The colour of the data points and error bars represents the different models. Bold letters and asterisks indicate significant predictors (*α* = 0.05, *p*-value < 0.05). Model coefficients correspond to the logarithm of odds ratios. Coefficients for chemical compounds and raised-body displays were higher in early contests, whereas the effects of type II foot shakes, residency status and prior contest history became more pronounced in later contests.

### (c) Scent-marks and residency in high-quality sites

The robust MANOVA detected no evidence of a multivariate difference in the scent-mark composition of males settled in high-quality versus low-quality sites or the rocks in between (Wald-type statistic; WTS = 28.00, d.f. = 20, *p* = 0.109), and this result was supported by parametric bootstrap resampling (*p* = 0.482 for WTS; *p* = 0.569 for modified ANOVA-type statistic; MATS). Accordingly, none of the examined chemical compounds showed significant differences among site quality categories in the robust one-way ANOVAs (supplementary material, table S13). Pooling rock and low-quality sites yielded qualitatively identical results. Although the asymptotic WTS was significant (WTS = 21.35, d.f. = 10, *p* = 0.019), this effect was not supported by parametric bootstrap resampling (*p* = 0.130 for WTS; *p* = 0.311 for MATS).

### (d) Scent-marks and fertilization success

Across analyses, the number of eggs fertilized by a male relative to other potential sires (i.e. fertilization success) was primarily driven by male–female spatial proximity, whether modelled as exact co-residency or as the parental site-quality combination (figure 4a). Ergosterol was the only compound associated with fertilization success, showing a negative effect, whereas all other compounds and dominance rank had weak support (relative importance values;RIV < 0.50) (supplementary material, table S14; figure 4b).

**Figure 4.**
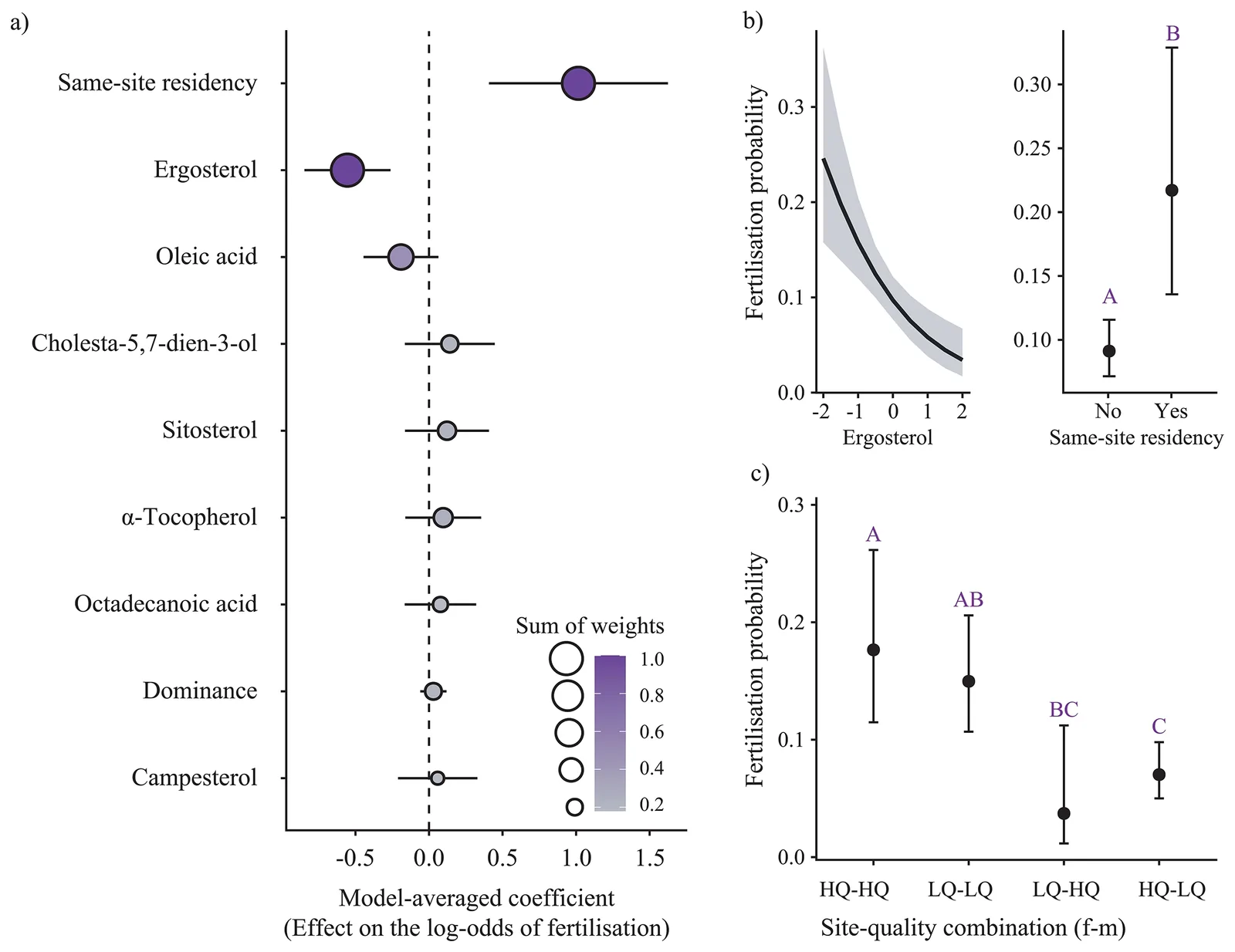
Predictors of per-egg fertilization success in males. (a) Forest plot of model-averaged conditional effects (points: effects on the log odds of fertilization; bars: ± CI95). Point size and colour reflect the summed model weights (SW). The analysis contrasts the effects of male–female spatial proximity and male traits (lipid abundances and dominance) on fertilization share. (b) Effect plots for predictors with CI95 excluding zero and SW > 0.55. Left: fertilization probability decreases with ergosterol (≈ 4% reduction per + 1 s.d.). Right: males sharing a site with the female show higher fertilization probability than males from other sites. (c) To test whether this effect depends on site quality, we included parental site-quality combinations. Low–low pairs performed similarly to high–high pairs, indicating that the same-site advantage is not restricted to high-quality dyads. Blue letters show Tukey-adjusted comparisons: shared letters indicate no significant differences.

Tukey-adjusted pairwise contrasts revealed that mixed-quality pairs (female–male: high-quality–low-quality or low-quality–high-quality) had markedly lower fertilization success compared with high-quality pairs (odds ratios (OR) 0.38 and 0.18, respectively; both *p* < 0.04). By contrast, low-quality pairs showed probabilities statistically indistinguishable from high-quality pairs (OR = 0.90, *p* = 0.99) and substantially higher than mixed-quality combinations (e.g. low-quality–low-quality vs high-quality–low-quality: OR = 2.36, *p* = 0.0048). Predicted fertilization probabilities reflected the same pattern: high-quality (0.18) and low-quality (0.15) pairs showed similarly high success, while high-quality–low-quality (0.07) and low-quality–high-quality (0.04) were consistently lower (figure 4c). The complete-case analyses (excluding males lacking chemical data) produced nearly identical qualitative results (supplementary material, table S15): same-site residency (std. *β* = 1.59) and ergosterol (std. *β* = –0.66) remained the only strong predictors, and the site-quality combinations showed the same hierarchy (high-quality–high-quality ≈ low-quality–low-quality > mixed-quality).

## 4. Discussion

This study provides a comprehensive empirical test of the links between male scent-mark composition and sexual selection in a lacertid lizard, integrating reproductive success and socio-spatial behaviour in semi-natural conditions. We show that scent-mark composition predicts male reproductive success indirectly through associations with socio-spatial dominance. By contrast, we found no evidence that females use male scent-mark composition for mate assessment. Reproductive success was unrelated to chemical compounds previously suggested to mediate female choice, aside from a negative association with ergosterol, and we detected no signature of cryptic female choice. Fertilization patterns were instead overwhelmingly shaped by spatial proximity, whereby females preferentially established themselves in high-quality territories and dominant resident males predominantly reproduced with them. Overall, our findings indicate that scent-marks function primarily in male–male competition and territorial dynamics, rather than female choice.

### (a) The role of scent-marks in male intrasexual competition

Residency in high-quality sites was the strongest predictor of male reproductive success (figure 2), and dominance determined which males acquired and retained these territories. High-quality territories offer improved basking conditions, refuge structure and proximity to females, resulting in reduced sperm competition and higher reproductive returns. Males in high-quality sites also mated with larger, more fecund females, resulting in higher reproductive returns per mating. These findings support the idea that competition for spatial resources, rather than chemically mediated female choice, defines the landscape of sexual selection for male *P. muralis* [9].

Consistent with prior work, our experiment supports a central role of chemical communication in male territorial behaviour [7]. We detected several links between scent-mark composition and socio-spatial dominance. Campesterol levels (tightly correlated with ergosta-5,8-dien-3-ol) were positively associated with residency in high-quality sites, and ergosterol showed a consistent negative association with dominance. After controlling for key determinants of contest outcome such as behavioural displays, prior contest history and residency status [4], our analyses revealed that males with high levels of cholesta-5,7-dien-3-ol and α-tocopherol, and low levels of ergosterol, were more likely to prevail in agonistic confrontations (figure 3). These associations were generally stronger in early contests than in later ones, whereas behavioural displays, residency and accumulated winning record grew in importance as territorial relationships crystallized. This dynamic is consistent with the idea that relatively static, condition-dependent compounds may function as quality signals early in territory establishment, whereas dynamic signals and ‘limited-war’ mechanisms—conveying information on motivation, identity and experience—become increasingly influential once dominance relationships and territories are established [73].

### (b) The role of scent-marks in female choice

Across multiple modelling approaches, we found no evidence that females favoured males with higher levels of α-tocopherol, oleic acid, cholesta-5,7-dien-3-ol or other compounds previously proposed as honest signals of male quality [10]. Moreover, most females settled in high-quality sites even though scent-mark composition did not differ between males occupying high-quality and low-quality sites. These results suggest that females prioritize high-quality habitat patches over male traits when choosing where to settle. Because females arrived in enclosures where males were already established, their settlement patterns provide a stringent test: if females were attracted to males’ chemical compounds, they should have preferentially settled near males with preferred scent-mark composition or biased fertilization toward such males. Instead, females aggregated in high-quality sites, and their eggs were primarily fertilized by males that co-settled with them—typically a minority of dominant males occupying these sites. Crucially, females in low-quality sites did not bias fertilizations towards males in high-quality sites or those with higher levels of the examined compounds, reinforcing that spatial proximity, rather than chemical assessment, determines fertilization outcomes (figure 4). Together, these findings weaken the case for a major role of quantitative variation in scent-mark lipid composition in female mate assessment. Instead, they are consistent with a socio-spatial system in which choosing high-quality sites may simultaneously bias females towards competitively successful males, thereby weakening selection for mate sampling and making spatial overlap a major determinant of mating opportunities [9,25,30,41].

Among the examined compounds, only ergosterol (vitamin D₂) showed a detectable direct association with relative mating success, with higher levels linked to fewer mates (figure 2a). However, this effect disappeared once the analysis was restricted to males with confirmed offspring (figure 2b), suggesting that the initial association originated from differential ergosterol levels between successful and unsuccessful reproducing males, rather than a quantitative relationship with relative mating success among successful males. In both analyses, ergosterol remained negatively related to male dominance, suggesting that any influence of this compound on fitness is indirect and mediated through territorial dynamics.

While females have been suggested to rely on scent-mark composition to indirectly assess territory quality [10,16], our results offer little support for this hypothesis. Females preferentially settled in high-quality sites despite dominant males there producing lower ergosterol and showing no elevated levels of any other examined compounds. A more parsimonious explanation is therefore that females assess habitat features directly [26] rather than relying on an indirect (possibly absent) relationship between male scent-marks and territory quality, as occurs in other vertebrates [32]. This interpretation is consistent with previous, largely observational evidence that female lizards preferentially settle according to resource quality rather than male phenotype [74], and our experiment provides experimental support for resource-based settlement under semi-natural conditions. However, a previous mesocosm experiment found that male, but not female, spatial distributions were associated with experimentally defined site quality [52], indicating that female responses to habitat-quality manipulations may depend on the spatial and social context of the experiment.

Support for the hypothesis that females use scent-mark composition for mate assessment is largely based on laboratory trials relying on preference proxies such as tongue-flick rates or time spent near odours. While differences in responses among males can indicate individual discrimination, such proxies do not necessarily imply mate assessment and may instead reflect recognition of, or attraction to, male-associated chemical cues [9,75]. For example, females of *Iberolacerta cyreni* show elevated tongue-flick responses to ergosterol, but responses barely exceed those elicited by natural male secretions [16], suggesting that ergosterol may act as a species-typical male cue rather than a quality signal. Similarly, female aggregation in ergosterol-enriched areas may reflect detection of male-occupied territories rather than preference for higher ergosterol levels [76]. In fact, lacertid females tend to prefer areas scented by adult males [77] and even areas scented by multiple males [78]. Such patterns are consistent with females being attracted to areas with conspecific scent-marks—often corresponding to high-quality sites—without necessarily discriminating among individual males [9,24,77]. Moreover, mate assessment can evolve only if it yields net fitness benefits to females [79]. In species lacking paternal care, such as lacertid lizards, these benefits are expected to be primarily indirect [80]. In lacertids, such benefits have been demonstrated in the context of genetic compatibility, including major histocompatibility complex (MHC)-associated preferences for male femoral gland secretions that enhance offspring viability [81–83]. However, whether females obtain comparable benefits by assessing quantitative variation in scent-mark lipids, as predicted under good-genes models of chemical signalling [14], remains unknown.

## 5. Conclusions

Research on chemical communication in lizards, and lacertids in particular, provides key insights into how scent-marks mediate behaviour and social interactions. However, the ultimate consequences in terms of fitness have remained speculative, largely owing to a lack of studies integrating scent-mark composition with behaviour and measuring its outcomes in natural settings. Here we show that a subset of scent-mark compounds correlates with male reproductive fitness via their association with socio-spatial dominance, whereas female settlement and fertilization patterns are driven by resource distribution and spatial proximity to males. This points to a role of scent-mark composition in territorial dynamics rather than female choice. In parallel with evaluating the functional consequences of scent-mark composition, its role in chemical communication also needs to be considered in terms of signal efficacy—that is, how compound properties affect the transmission, persistence and detectability of scent-marks in natural environments [84,85]. Further progress in this field will require integrative approaches linking chemical composition with perception, physiology, signal efficacy and the spatial ecology of scent deposition [86,87].

## Supplementary Material



## Data Availability

The data and code supporting this article are available at the following Zenodo repository: [88]. Supplementary material is available online [89].
