# Supplementary material for: Male scent-marks predict fitness via socio-spatial dominance, but not female choice, in a lacertid lizard
Source: Proc Biol Sci. 2026 Nov 4;293(2082):20260863. doi: 10.1098/rspb.2026.0863 (PMC13730803; doi:10.1098/rspb.2026.0863)
Supplement: Supplementary file 1 [file rspb.2026.0863.zip › rspb.2026.0863/rspb20260863_si_001.docx]

**Appendix S1: Supplementary material**

**Title: Male scent-marks predict fitness via socio-spatial dominance, but not female choice, in a lacertid lizard**

**Authors:** Abalos, Javier* ^1,2^; Bartolomé, Alicia ^1^; Pérez i de Lanuza, Guillem ^1^; Aubret, Fabien ^4,5^; Uller, Tobias ^3^; Carazo, Pau ^1^; Font, Enrique ^1^; García-Roa, Roberto* ^1^

**Affiliations:** *^1^ Ethology Lab, Instituto Cavanilles de Biodiversidad y Biología Evolutiva, Universitat de València, València, Spain. ^2^ School of Biological Sciences, University of Tasmania, Hobart, Australia. ^3^ Department of Biology, Lund University, Lund, Sweden. ^4^ SETE, Station d’Ecologie Théorique et Expérimentale, UMR5321, Centre National de la Recherche Scientifique, 2 Route du CNRS, 09200 Moulis, France. ^5^ Charles Stuart University, 7 Major Innes Rd, Port Macquarie Campus, 2444, NSW, Australia*

**Journal:** *Proceedings of the Royal Society B: Biological Sciences*

**Article DOI:** 10.1098/rspb.2026.0863

**Contents:**

1. Expanded material and methods
   1.1. Scent mark composition analyses
   1.2. Preprocessing of scent mark composition data
   1.3. Behavioural observations
   1.4. Geographic variation in scent mark composition
   1.5. Spatial behaviour analysis
   1.6. Parentage analyses
   1.7. Piecewise structured equation modelling
   1.8. Bradley-Terry models on male-male competition
   1.9. Model fitting
2. Expanded results
   2.1. Scent mark composition analyses
   2.2. Geographic variation in scent mark composition
   2.3. Socio-spatial behaviour and fitness

3.	References

4.	Tables:

Table S1- Table S13.

5.	Figures:

Figure S1- Figure S5.

1. **Expanded materials and methods**
   1.1. *Female housing and hibernation before the experiment*

To ensure captured females were not gravid, we captured females at the end of the breeding season (September 5 2017) and transferred them to the Station d’Ecologie Théorique et Expérimentale (SETE, Moulis, France). There, we housed females in groups of 3-5 coming from the same locality in outdoor circular plastic tubs (170 cm diameter, 60 cm high), where they were kept for 130 days. In May 2018, after an artificial hibernation period, we elevated the temperature to 10ºC for one week and then returned the females in the outdoor tubs for another week while we captured the males.

We installed an anti-bird net covering each of the 33 tanks used, which prevented avian predators while allowing local invertebrates to get inside the tanks and serve as primary food source. To increase habitat complexity, we complemented the naturally occurring vegetation inside the tanks by providing a perforated brick to serve as shelter, two wood logs, and a water dish to collect rain-water. We visited the tanks monthly to trim the weeds and provide an additional supply of food (*Tenebrio molitor* larvae and *Acheta domesticus* adults). On 14 February 2018, we re-captured the females within the outdoor tanks, installed them in individual hibernation pods consisting of a plastic tub (12 cm diameter, 8 cm high) covered with a perforated lid and filled with moist coco husk, and stored them for 10 weeks in a dark climatic chamber to mimic the conditions at their capture 17 sites (1st week: 10ºC/ 2nd-9th weeks: 5ºC/ 10th week: 10ºC).

1.2. *Scent mark composition analyses*

Femoral secretions were extracted from the left hindlimb of each *P. muralis* male following standardised protocols [1,2]. Samples were obtained by gently pressing around the femoral pores and collecting the resulting secretion in glass vials (1.1 ml). Blank control vials were included throughout the process to monitor potential contamination during handling. To minimise sample degradation, all vials were stored at −20 °C. In the lab, samples were removed from the vials using clean tweezers, weighed in disposable micro aluminium dishes (±0.0001 mg), and processed at a constant room temperature of 20°C. All equipment was cleaned with n-hexane (99%) before and after each measurement.

For a general characterisation and semiquantitative analysis of the chemical composition of male scent marks, each sample was treated with 100 μl of hexane containing 1 μg ml⁻¹ of n-docosane D46 as an internal standard (IS). This internal standard allowed compound responses to be compared independently [2], which is crucial when examining inter-individual variation in compound abundance. Samples were agitated for 15 minutes in an orbital shaker, and the resulting extract was transferred to a conical glass insert using a Pasteur pipette for immediate analysis. Chemical analyses were conducted using gas chromatography–mass spectrometry (GC–MS). We used an Agilent 6890N gas chromatograph equipped with an HP-5MS UI capillary column (30 m × 0.25 mm × 0.25 μm), coupled to a TSQ Quantum XLS triple quadrupole mass spectrometer for quantification. A 1 μl aliquot of each extract was injected via an Agilent 7683B auto-injector into a 270 °C inlet in split-less mode (with the split valve opened after 2 minutes). The oven temperature programme was: 50 °C for 1 minute, then ramped at 40 °C min⁻¹ to 150 °C, followed by 5 °C min⁻¹ to 320 °C, and held for 9 minutes. Helium was used as the carrier gas at a constant flow rate of 1.4 ml min⁻¹. Mass spectra were acquired in scan mode (m/z 33–450) using electron impact ionisation at 70 eV. The ion source and quadrupole temperatures were set to 150 °C and 230 °C, respectively, and the transfer line was maintained at 300 °C. Compound identification followed standard procedures widely used in chemical studies of European lizards [3–5], using: (1) retention time comparisons with available authentic standards analysed under similar conditions, and (2) mass spectral matches against the NIST 2017 library using the Mass Spectral Search Program. A compound was considered positively identified when the match score exceeded 80%. All analyses were conducted at the Advanced Research Facilities of the University of the Basque Country (SGIker, UPV–EHU, Spain).

While this methodology provides a tentative broad overview of scent mark composition, it is not optimal for the fine-scale identification and quantification of target compounds (see **Section 2.1.**, **Table S2**, and **Fig S1** for comparison between scan and targeted analyses). Therefore, we implemented a second step focused on specific compounds of interest: cholesterol, α-tocopherol, ergosterol (provitamin D2), oleic acid, hexadecanoic acid, octadecanoic acid, campesterol, cholesta-5,7-dien-3-ol, and ergosta-5,8-dien-3-ol. These compounds were selected on the base of their alleged role in sexual selection according to the literature (reviewed in [6]. Using the mass spectra of each compound, we used diagnostic fragment ions (m/z) that allowed us to confirm their presence in scan-mode chromatograms. Then, we extracted their signal intensities and retention times (tR) from each sample (i.e. set of fragment ions) to confirm compound identity and quantify relative abundance.

1.3. *Preprocessing of scent mark composition data*

Graphical exploration of the data from the targeted analysis of scent mark composition revealed a pronounced positive skew across all examined compounds, characterized by an excess of low-value readings relative to a normal distribution. Additionally, many compounds exhibited both positive skew and extreme high-value observations (outliers), complicating statistical analyses. To mitigate the impact of these outliers, Winsorization was applied to all compound levels, capping values at the 95th percentile (**Fig. S2**). To address zeros across varying scales (which is essential for logarithmic transformations), we applied adaptive pseudo-count addition by identifying the smallest nonzero value for each compound and adding 10% of across individuals [7,8]. We then applied a logarithmic transformation to attenuate skewness in the data. Finally, to enable comparisons across variables with differing scales, z-scores were computed by standardizing each variable to have a mean of zero and a standard deviation of one.

We found considerable levels of covariation between the ten examined compounds, with α-tocopherol and oleic acid showing the highest levels of independent variation (**Fig. S3**). To reduce problems resulting from multicollinearity in statistical modelling we used the *findCorrelation* function in the caret R package to select a subset of compound concentration variables showing no pairwise Pearson correlation coefficient higher than ± 0.6 [9]. This resulted in keeping seven out of ten examined compounds (i.e. cholesta-5,7-dien-3-ol, ergosterol, octadecanoic acid, campesterol, oleic acid, α-tocopherol, and sitosterol). The three excluded compounds are well represented by variation in other retained compounds (**Fig. S3**). More specifically, ergosta-5,8-dien-3-ol is well represented by ergosterol, cholesterol is well represented by variation in cholesta-5,7-dien-3-ol, and hexadecanoic acid is well represented by variation in octadecanoic acid (**Table S3**).

1.4. *Geographic variation in scent mark composition*

As a sensitivity analysis, we tested whether overall scent-mark composition differed among the 12 general capture areas using a PERMANOVA based on Euclidean distances of the semiquantitative compound abundances (9999 permutations).

1.5. *Behavioural observations*

From May 23 to June 22, we conducted observations of spatial and social behaviour during peak activity hours (9.00-19.30), waiting at least 1 h between consecutive visits to the same enclosure. One observer performed sequential rounds visiting all the enclosures every 2.5 h to collect data on the lizards’ spatial behaviour (i.e. positional data). Using scan sampling, we determined the identity and location of every lizard in sight on a scale map of the enclosure that included the six wooden pallets. To balance sampling effort across enclosures, scanning of a single enclosure was restricted to a maximum period of 15 min after the first lizard was spotted. Meanwhile, two researchers recorded the identity, position and behaviours of the lizards participating in social interactions using a behaviour sampling rule in recording sessions lasting 40 min. A social interaction was considered to occur whenever a marked lizard in our visual range directed any of the behaviours listed in **Table S4** toward a conspecific. Consecutive interactions involving the same lizards were recorded as different events whenever the participants remained further than 30 cm apart for longer than 2 min. Non-independence due to repeated individuals and prior contest experience was addressed statistically (see *1.8.* *Bradley-Terry models on male-male competition*). For further details, see [10,11].

1.6. *Spatial behaviour analysis*

We analyzed positional data using kernel density estimation (KDE) as implemented in the R package adehabitatHR [12,13], with spatial objects handled using the sp package [14]. To determine an appropriate smoothing parameter (h) for the kernel density estimates, we initially applied the least-squares cross-validation (LSCV) method (h = "LSCV"). This method minimizes the mean integrated squared error by evaluating the prediction error of each point using leave-one-out cross-validation. Although some individual models failed to converge using LSCV, we successfully obtained optimal h values for a majority of individuals (*N* = 161). We calculated the mean LSCV-derived h across converged models, which yielded an average value of approximately 0.24 (± 0.17 SD). Based on this result, and to maintain consistency and comparability across individuals, we used a fixed bandwidth of h = 0.2 for all kernel estimations. Using this common smoothing parameter, we estimated utilization distributions (UDs) for each lizard with the kernelUD() function and extracted the 95% and 50% probability contours using the getverticeshr() function. These contours were interpreted as the individual's home range and core area, respectively. Each lizard was assigned residency at a specific site based on the location of peak density within the core area.

1.7. *Parentage analyses*

Parentage was assigned using multilocus microsatellite genotypes [15,16] and likelihood-based methods [17,18]. We isolated DNA from tail-tip samples using the DNeasy 96 Blood & Tissue Kit (Qiagen, Valencia, CA, USA), obtaining a final elution volume of 150 μl in AE buffer. We then combined the primers of six microsatellite loci described in *P. muralis* [15,16] into two different multiplexes (MPA: Pm16, Pm09, PmurC168; MPB: Pm19, Pm14, PmurC038) and ran standard PCR with 26 cycles and a final extension step of 30 min at 60°C. Forward primers were labeled with different fluorescent dyes (FAM, NED, HEX). Diluted PCR products (1:5) were genotyped together with an internal ladder (Red ROX-500) on an ABI 3130 genetic analyzer (Applied Biosystems Inc.). One researcher scored the alleles for every adult and juvenile lizard in Geneious 7.0.4 (Biomatters, available at http://www.geneious.com), which we used to conduct parentage analysis in Cervus 3.0 [17,18]. We assigned paternity based on the log-likelihood statistic of each mother–father–offspring trio (LOD scores), using two confidence levels (strict: 95%, relaxed: 80%) and the nine males within each enclosure as candidate fathers. Critical LOD scores were determined by running a simulation paternity analysis based on 100,000 offspring with known mothers and nine candidate fathers. We could reliably assign paternity to every offspring examined (strict: 208 juveniles, relaxed: 230 juveniles).

1.8. *Piecewise structured equation modelling*

Piecewise SEM is a form of path analysis particularly suited for evaluating direct and indirect relationships among multiple interrelated variables in complex systems [19]. This approach allows for flexible model specification, including random effects and non-Gaussian error distributions, making it well-suited for hierarchical data with complex dependency structures [20]. To avoid problems of reduced statistical power and overparameterization, we first examined the relationships among potential predictor variables using a Spearman correlation matrix, calculated with the *chart.Correlation* function from the PerformanceAnalytics package in R [21]. Spearman's rank correlation was used because it is robust to non-normal distributions and non-linear relationships. To control for the false discovery rate across multiple correlation tests, we adjusted *P*-values using the *p.adjust* function with the Benjamini–Yekutieli procedure [22] implemented in the stats package. We explored the relationship among dominance rank index (DRI), core range size, number of females courted (tail-grabs), number of females guarded (co-perching), co-perchings per female (CP/F), HQ residency (HQr; HQ = 1, LQ or rocks = 0), relative mating success (RMS), sperm competition intensity (SCI), and relative reproductive success (RRS) (**Fig. S4**).

The initial pSEM (**Fig. S5**) specified direct effects of dominance (DRI), habitat residency (HQr), co-perching per female (CP/F), and relative mating success (RMS) on relative reproductive success (RRS). Direct effects of DRI, HQr, and CP/F on RMS and SCI were also included. Male chemical traits and morphology (mass, head-length residuals) were modelled as predictors of DRI, HQr, and CP/F. Mass and HL res were selected as morphological predictors because lizards were paired within each enclosure to minimize variation in SVL differences, and BCI was highly correlated with mass (**Fig. S3**). GLMMs for RMS, RRS, and SCI were fitted using a negative binomial error distribution to account for overdispersion, while the model for DRI was fitted as a linear mixed model (LMM) with a Gaussian error distribution, and the model for HQr used a binomial error distribution.

1.9. *Bradley-Terry models on male-male competition*

Individuals with missing data for specific predictors were handled using the null model framework, which assigns separate ability scores [23,24]. For each male, the first contest was assigned a prior contest outcome value of 0, as no individual had previous wins [25,26]. Repeated encounters between the same dyads were generally well separated in time: across 315 intervals between consecutive encounters, the median elapsed time was 2 d 22 h (IQR = 4 h 26 min–7 d 2 h), with only 8.9% occurring within 10 min.

Model selection was conducted using backwards single term deletions (*P-value* < 0.05) of the saturated model followed by model comparisons via likelihood ratio tests (at α = 0.05) using the function *drop1* included in base R [27]. Day nine was chosen as a cutoff point between early and late contests based on a marked increase in male–male confrontations observed thereafter [11].

1.10. *Model fitting*

All models were fitted using functions available in the glmmTMB and lme4 packages of R [28]. All numerical variables were centered and scaled before running the models [29]. To account for the mesocosm design of the experiment, we included the experimental cell as a random factor in all GLMM models. We graphically explored that residuals conformed to homoscedasticity and normality assumptions using the function *simulateResiduals* from the package DHARMa [30]. We also tested for zero-inflation, overdispersion and collinearity using functions available in the DHARMa and Performance packages in R [30,31]. Model selection was conducted using the corrected Akaike’s information criterion (AICc) [32]. As model selection methods for piecewise SEMs do not yet accommodate the full set of models nested within a saturated structure, for the pSEM procedure we conducted model selection using backwards single term deletions of the saturated model until we obtained the model showing the lowest value of AICc [33,34]. Because the piecewiseSEM package does not provide measures of uncertainty for standardized path coefficients, we complemented our analysis by using the *semEff* function from the homonym R package [35]. This function summarizes the direct, indirect, and total effects in the SEM and estimates bootstrapped CI_95_ for standardized coefficients. Importantly, these estimates are computed on a different scale than the standardized coefficients returned by *coefs* in piecewiseSEM, and thus are not directly comparable in magnitude. Bootstrapped estimates were obtained using *bootEff* with 1,000 replicates, incorporating the random effects structure of the original models.

To further refine inference on individual pathways, we complemented the pSEM-based selection with an exhaustive model selection approach for each of its component models, implemented via the *dredge* function in the MuMIn package [36]. This same method for model selection was applied to beta-binomial mixed models on fertilisation success. We considered models with AICc differences (ΔAICc) < 4 to have substantial support, extending the conventional threshold of ΔAICc < 2 to better capture alternative plausible models [27,32,37]. We examined multiple comparisons among factor levels in the top-ranked models using estimated marginal means (EMMs), computed with the *emmeans* function in the homonym R package [38]. Pairwise contrasts were performed with Tukey-adjusted *p*-values to account for multiple comparisons [39]. We calculated the model averaged coefficients and 95% confidence intervals (CI) along with the relative importance of variables (RIV) for each variable by summing the normalized AIC_C_ weights of the models in this subset where a given variable was present. We considered a variable to be biologically significant if its CI_95_ did not include 0 and it had RIV ≥ 0.55 [34]. All analysis were conducted in R v.4.2.2. [40].

2. **Expanded results**
   2.1. *Scent mark composition analyses*

Compared to results from the scan analysis, the targeted methodology resulted in a much lower number of blank readings, with the exception of α-Tocopherol and Oleic acid (**Table S2**). To compare results from the scan and the targeted analysis, we plotted against each other the corresponding individual readings obtained in both analyses for the nine compounds present in both results. Blank readings from the scan analysis were substituted with zeroes, while blank readings in the targeted analysis were left as NA. To deal with deviations from normality, we added a constant of 0.01 to all values and then extracted the corresponding logarithm. Graphical examination of the resulting scatterplots reveals that blank readings in the scan analysis did not generally correspond to low concentration levels in the targeted analysis, arguing against the common practice of substituting blank readings from scan analysis with zeroes (**Fig. S1**). Consequently, we deemed unreliable the results from the scan analysis and based all subsequent analysis on results from the targeted methodology, where blank readings can confidently be assumed to reflect low concentration levels.

2.2. *Geographic variation in scent mark composition*

Overall scent-mark composition differed significantly among capture areas (PERMANOVA, *R*² = 0.15, *P* = 0.020), indicating modest geographic variation in chemical profiles. However, geographic origin did not explain male dominance, home-range size, relative mating success or relative reproductive success (all *P* > 0.4), suggesting that geographic variation was not associated with subsequent behavioural or reproductive performance.

2.3. *Socio-spatial behaviour and fitness*

Spearman correlation analyses revealed several significant associations between male dominance, socio-spatial behaviour, and male fitness (**Fig. S4**). Dominance rank was negatively associated with core range size (ρ = –0.45) and sperm competition intensity (ρ = –0.13), but positively with the number of females guarded (co-perching; ρ = 0.35), the number of females courted (tail-grab; ρ = 0.46), and the probability of settling in a HQ site (ρ = 0.44). These patterns suggest that dominant males had more restricted ranges, encountered more females, and faced reduced sperm competition intensity, overall showing higher relative mating (ρ = 0.20) and reproductive success (ρ = 0.27). Core range size was negatively correlated with both average sperm competition intensity (ρ = –0.47) and relative reproductive success (ρ = –0.44), while being positively related to relative mating success (ρ = 0.27). The number of females guarded showed strong positive correlations with number of females courted (ρ = 0.31) and both mating (ρ = 0.27) and reproductive success (ρ = 0.27). Expectedly, relative mating success was highly positively associated with reproductive success (ρ = 0.82) and negatively with average sperm competitors (ρ = -0.51), confirming that exclusive access to multiple mates is a key determinant of male fitness in this system.

3. **References**

1.	Ibáñez A, Menke M, Quezada G, Jiménez-Uzcátegui G, Schulz S, Steinfartz S. 2017 Diversity of compounds in femoral secretions of Galápagos iguanas (genera: Amblyrhynchus and Conolophus), and their potential role in sexual communication in lek-mating marine iguanas (Amblyrhynchus cristatus). *PeerJ* **5**, e3689. (doi:10.7717/peerj.3689)

2.	García-Roa R, Sáiz J, Gómara B, López P, Martín J. 2018 How to tackle chemical communication? Relative proportions versus semiquantitative determination of compounds in lizard chemical secretions. *Ecology and Evolution* **8**, 2032–2040. (doi:10.1002/ece3.3825)

3.	García-Roa R, Sáiz J, Gómara B, López P, Martín J. 2017 Dietary constraints can preclude the expression of an honest chemical sexual signal. *Sci Rep* **7**, 6073. (doi:10.1038/s41598-017-06323-8)

4.	Donihue CM, Herrel A, Martín J, Foufopoulos J, Pafilis P, Baeckens S. 2020 Rapid and repeated divergence of animal chemical signals in an island introduction experiment. *Journal of Animal Ecology* **89**, 1458–1467. (doi:10.1111/1365-2656.13205)

5.	Baeckens S, Martín J, Pafilis P, Herrel A, Donihue CM. 2025 Temporal dynamics of scent mark composition in field-experimental lizard populations. *Functional Ecology* **39**, 154–164. (doi:10.1111/1365-2435.14687)

6.	Martín J, López P. 2015 Condition-dependent chemosignals in reproductive behavior of lizards. *Hormones and Behavior* **68**, 14–24. (doi:10.1016/j.yhbeh.2014.06.009)

7.	Martín J, López P. 2014 Pheromones and Chemical Communication in Lizards. In *Reproductive Biology and Phylogeny of Lizards and Tuatara*, CRC Press.

8.	Chen L, Reeve J, Zhang L, Huang S, Wang X, Chen J. 2018 GMPR: A robust normalization method for zero-inflated count data with application to microbiome sequencing data. *PeerJ* **6**, e4600. (doi:10.7717/peerj.4600)

9.	Kuhn, Max. 2008 Building Predictive Models in R Using the caret Package. *Journal of Statistical Software* **28**, 1–26. (doi:10.18637/jss.v028.i05)

10.	Abalos J, Pérez i de Lanuza G, Bartolomé A, Liehrmann O, Laakkonen H, Aubret F, Uller T, Carazo P, Font E. 2020 No evidence for differential sociosexual behavior and space use in the color morphs of the European common wall lizard (Podarcis muralis). *Ecology and Evolution* **10**, 10986–11005. (doi:10.1002/ece3.6659)

11.	Abalos J, Pérez i de Lanuza G, Bartolomé A, Liehrmann O, Aubret F, Font E. 2024 Behavioral threat and appeasement signals take precedence over static colors in lizard contests. *Behavioral Ecology* **35**, arae045. (doi:10.1093/beheco/arae045)

12.	Calenge C. 2006 The package “adehabitat” for the R software: A tool for the analysis of space and habitat use by animals. *Ecological Modelling* **197**, 516–519. (doi:10.1016/j.ecolmodel.2006.03.017)

13.	Calenge C, Fortmann-Roe contributions from S. 2023 *adehabitatHR: Home Range Estimation*. See https://CRAN.R-project.org/package=adehabitatHR.

14.	Pebesma EJ, Bivand RS. 2005 Classes and methods for spatial data in R. *R News* **5**, 9–13.

15.	Richard M, Stevens VM, Hénanff ML, Coulon A, Lourdais O, Clobert J. 2012 Fourteen new polymorphic microsatellite loci for the wall lizard *Podarcis muralis* (Sauria: Lacertidae). *Molecular Ecology Resources* **12**, 374–376.

16.	Heathcote RJP, Dawson DA, Uller T. 2014 Characterisation of nine European wall lizard (*Podarcis muralis*) microsatellite loci of utility across sub-species. *Conservation Genetics Resources* **7**, 85–87. (doi:10.1007/s12686-014-0294-5)

17.	Marshall TC, Slate J, Kruuk LEB, Pemberton JM. 1998 Statistical confidence for likelihood‐based paternity inference in natural populations. *Molecular Ecology* **7**, 639–655. (doi:10.1046/j.1365-294x.1998.00374.x)

18.	Kalinowski ST, Taper ML, Marshall TC. 2007 Revising how the computer program cervus accommodates genotyping error increases success in paternity assignment. *Molecular Ecology* **16**, 1099–1106. (doi:10.1111/j.1365-294X.2007.03089.x)

19.	Grace JB, Anderson TM, Olff H, Scheiner SM. 2010 On the specification of structural equation models for ecological systems. *Ecological Monographs* **80**, 67–87. (doi:10.1890/09-0464.1)

20.	Lefcheck JS. 2016 piecewiseSEM: Piecewise structural equation modelling in r for ecology, evolution, and systematics. *Methods in Ecology and Evolution* **7**, 573–579. (doi:10.1111/2041-210X.12512)

21.	Peterson BG *et al.* 2018 Package ‘performanceanalytics’. *R Team Cooperation* **3**, 13–14.

22.	Benjamini Y, Yekutieli D. 2001 The control of the false discovery rate in multiple testing under dependency. *https://doi.org/10.1214/aos/1013699998* **29**, 1165–1188. (doi:10.1214/AOS/1013699998)

23.	Stapley J, Whiting MJ. 2006 Ultraviolet signals fighting ability in a lizard. *Biology letters* **2**, 169–172. (doi:10.1098/rsbl.2005.0419)

24.	Firth D, Turner HL. 2012 Bradley-Terry models in R : the BradleyTerry2 package. *Development* , 1–10. (doi:10.18637/jss.v048.i09)

25.	Stuart-Fox DM, Firth D, Moussalli A, Whiting MJ. 2006 Multiple signals in chameleon contests: designing and analysing animal contests as a tournament. *Animal Behaviour* **71**, 1263–1271. (doi:10.1016/j.anbehav.2005.07.028)

26.	Kar F, Whiting MJ, Noble DWA. 2016 Influence of prior contest experience and level of escalation on contest outcome. *Behavioral Ecology and Sociobiology* **70**, 1679–1687. (doi:10.1007/s00265-016-2173-4)

27.	Symonds MRE, Moussalli A. 2011 A brief guide to model selection, multimodel inference and model averaging in behavioural ecology using Akaike’s information criterion. *Behavioral Ecology and Sociobiology* **65**, 13–21. (doi:10.1007/s00265-010-1037-6)

28.	Brooks ME, Kristensen K, van Benthem KJ, Magnusson A, Berg CW, Nielsen A, Skaug HJ, Mächler M, Bolker BM. 2017 glmmTMB balances speed and flexibility among packages for zero-inflated generalized linear mixed modeling. *R Journal* **9**, 378–400. (doi:10.32614/rj-2017-066)

29.	Schielzeth H. 2010 Simple means to improve the interpretability of regression coefficients. *Methods in Ecology and Evolution* **1**, 103–113. (doi:10.1111/j.2041-210X.2010.00012.x)

30.	Hartig F. 2024 DHARMa: Residual Diagnostics for Hierarchical (Multi-Level / Mixed) Regression Models. (doi:10.32614/CRAN.package.DHARMa)

31.	Lüdecke D, Makowski D, Waggone P. 2020 performance: Assessment of Regression Models Performance. R package version 0.4.4., https://CRAN.R-project.org/package=performance.

32.	Burnham KP, Anderson DR. 2004 Multimodel Inference: Understanding AIC and BIC in Model Selection. *Sociological Methods & Research* **33**, 261–304. (doi:10.1177/0049124104268644)

33.	Shipley B. 2013 The AIC model selection method applied to path analytic models compared using a d-separation test. *Ecology* **94**, 560–564. (doi:10.1890/12-0976.1)

34.	Garrido M, Hansen SK, Yaari R, Hawlena H. 2022 A model selection approach to structural equation modelling: A critical evaluation and a road map for ecologists. *Methods in Ecology and Evolution* **13**, 42–53. (doi:10.1111/2041-210X.13742)

35.	Murphy MV. 2024 *semEff: Automatic Calculation of Effects for Piecewise Structural Equation Models*. (doi:10.32614/CRAN.package.semEff)

36.	Barton K. 2022 MuMIn: Multi-Model Inference. *R package version 1.46.0.*

37.	Richards SA. 2005 Testing Ecological Theory Using the Information-Theoretic Approach: Examples and Cautionary Results. *Ecology* **86**, 2805–2814. (doi:10.1890/05-0074)

38.	Midway S, Robertson M, Flinn S, Kaller M. 2020 Comparing multiple comparisons: practical guidance for choosing the best multiple comparisons test. *PeerJ* **8**, e10387. (doi:10.7717/peerj.10387)

39.	Lenth R. 2023 emmeans: Estimated marginal means, aka least-squares means. R package version 1.7. 2.

40.	R Core Team. 2025 *R: A Language and Environment for Statistical Computing*. Vienna, Austria: R Foundation for Statistical Computing. See https://www.R-project.org/.

4. **Tables**

**Table S1.** Geographic origin of the 180 lizards used in the experiment. Lizards were collected from 12 general areas, subdivided into 20 distinct capture localities according to the patchy distribution of the lizards across suitable habitat. Several of the capture localities contained lizards separated by more than 300 m, allowing individuals originating from the same capture locality to be assigned to the same enclosure while still satisfying our criterion of spatial independence. The table provides the coordinates of each capture locality together with the number of males and females collected.

| **Area** | **Capture locality** | **Latitude** | **Longitude** | **Males** | **Females** | **Total** |
| --- | --- | --- | --- | --- | --- | --- |
| Area 1 | ALP | 42.3782 | 1.8909 | 6 | 1 | 7 |
| Area 2 | ANGOSUTRINE | 42.4776 | 1.9524 | 7 | 13 | 20 |
| Area 2 | ANGOSUTRINE | 42.4851 | 1.9549 | 1 | 0 | 1 |
| Area 3 | CEMDOR | 42.4876 | 1.9471 | 7 | 5 | 12 |
| Area 3 | DORRES | 42.4845 | 1.9332 | 8 | 8 | 16 |
| Area 4 | CEREJA | 42.4759 | 1.9691 | 6 | 0 | 6 |
| Area 4 | LLIVIA | 42.4589 | 1.9808 | 5 | 5 | 10 |
| Area 5 | CORTVASSILL | 42.4942 | 1.8514 | 10 | 3 | 13 |
| Area 6 | FILATURE | 42.4870 | 1.9645 | 6 | 6 | 12 |
| Area 6 | MORRENA | 42.4919 | 1.9601 | 0 | 10 | 10 |
| Area 7 | ST MARTI | 42.4242 | 1.9017 | 0 | 2 | 2 |
| Area 7 | GER | 42.4113 | 1.8448 | 7 | 0 | 7 |
| Area 8 | LATOUR | 42.4626 | 1.8970 | 8 | 12 | 20 |
| Area 8 | LATOUR2 | 42.4630 | 1.8894 | 8 | 0 | 8 |
| Area 8 | LATOUR3 | 42.4693 | 1.8776 | 5 | 1 | 6 |
| Area 9 | PUIGCERDÁ | 42.4343 | 1.9313 | 6 | 0 | 6 |
| Area 10 | QUEROL | 42.4984 | 1.8437 | 0 | 1 | 1 |
| Area 11 | TARGAS | 42.4996 | 1.9964 | 0 | 5 | 5 |
| Area 11 | THEMIS | 42.5009 | 1.9701 | 0 | 10 | 10 |
| Area 12 | TUBERIA | 42.4985 | 1.9617 | 0 | 8 | 8 |

**Table S2.** Chemical compounds (lipidic fraction) in the femoral secretions of 78 *Podarcis muralis* males, ranked in descending order according to their prevalence of blank readings in the sweep analysis.

|  | Scan analysis | | Targeted analysis | |
| --- | --- | --- | --- | --- |
| Variable | **Blank** | **Proportion** | **Blank** | **Proportion** |
| Cholesterol | 1 | 0.013 | 1 | 0.013 |
| Cholesta-5,7-dien-3-ol | 11 | 0.141 | 0 | 0.000 |
| Squalene | 12 | 0.154 |  |  |
| α-Tocopherol | 15 | 0.192 | 32 | 0.410 |
| Docosemide13 | 19 | 0.244 |  |  |
| Hexadecanoic acid | 22 | 0.282 | 15 | 0.192 |
| Ester acid | 27 | 0.346 |  |  |
| Ester – no id | 27 | 0.346 |  |  |
| Octadecanoic acid-9-ester | 31 | 0.397 | 1 | 0.013 |
| Ergosta-5,7,2,5-trienol | 32 | 0.41 |  |  |
| Campesterol | 32 | 0.41 | 9 | 0.115 |
| Ergosta-5,8-dien-3-ol | 36 | 0.462 | 1 | 0.013 |
| Ergosterol | 37 | 0.474 | 0 | 0.000 |
| No id | 39 | 0.5 |  |  |
| Alkene | 42 | 0.538 |  |  |
| Oleic acid | 44 | 0.564 | 47 | 0.603 |
| Cholesta-4,6-dien-3-one | 44 | 0.564 |  |  |
| Cholesta-3,5-diene | 48 | 0.615 |  |  |
| No id | 51 | 0.654 |  |  |
| Alkene | 53 | 0.679 |  |  |
| Hexadecanal | 54 | 0.692 |  |  |
| No id | 56 | 0.718 |  |  |
| Octadecanoic acid | 63 | 0.808 |  |  |
| No id | 70 | 0.897 |  |  |
| No id | 72 | 0.923 |  |  |
| Sitosterol | - | - | 6 | 0.077 |

**Table S3.** Pearson correlation coefficients and *P-value*s corresponding to the compound concentration variables dropped from the analysis due to them being well represented by other retained variables (Pearson R > 0.6). Correlation coefficients were estimated after winsorization, adaptive pseudocount addition, logarithmic transformation, and Z-transformation.

| **Retained_Variable** | **Correlated_Variable** | **Correlation** | ***P-value*** |
| --- | --- | --- | --- |
| Cholesta-5,7-dien-3-ol | Cholesterol | 0.633 | 4.83E-10 |
| Ergosterol | Ergosta-5,8-dien-3-ol | 0.694 | 1.89E-12 |
| Octadecanoic acid | Hexadecanoic acid | 0.643 | 2.18E-10 |

**Table S4.** Partial ethogram of *Podarcis muralis* including social behaviours used to collect data on interactions during behavioural observations within the experimental enclosures.

| **Behaviour** | **Description** |
| --- | --- |
| Approach* | Movement toward a non-fleeing conspecific |
| Raised-body display | Gular extension, back-arching, shoulders raised, head down, sagittal compression (any combination) |
| Bite | One or more bites to another individual (excluding tail grab) |
| Retreat* | Movement away from a non-chasing conspecific |
| Chase | Rapidly following another FLEEING lizard |
| Flight | Fast-paced movement to withdraw from a CHASING lizard |
| Type II foot shakes | Rapid large amplitude vertical movements of forelimbs (belly down, head up posture), often accompanied by TAIL WAVE/SHAKE |
| Tail grab | A male bites the tail or inguinal region of a female. Often followed by copulation |
| Tail wave/shake | Vibrating entire tail (or its distal portion) swiftly from side to side |
| Mating | Two lizards engage in copulation |
| Co-perching | Two or more lizards lying together in close vicinity (<15 cm; >30 s) |
| * We classified the mode of locomotion used as either running (fast-paced) or any other mode of locomotion (slow-paced). | |

**Table S5**. Model selection results from the binomial GLMM assessing sex differences on the probability of settling in high-quality (HQ) versus low-quality (LQ) sites. Model # refers to the model ID used for reference. Fixed effects included in each model are indicated by their estimated coefficients (Intercept, Mass, Sex). df is the number of estimated parameters, AICc is the corrected Akaike Information Criterion, ΔAICc indicates the difference in AICc between model *i* and the top-ranked model, and Weight represents the relative probability that model *i* is the best model given the data.

| **Model #** | **Intercept** | **Mass** | **Sex** | **df** | **AICc** | **ΔAICc** | **Weight** |
| --- | --- | --- | --- | --- | --- | --- | --- |
| 3 | -0.09 |  | + | 3 | 208.3 | 0.00 | 0.625 |
| 4 | -1.19 | 0.18 | + | 4 | 209.3 | 1.02 | 0.375 |
| 2 | 1.48 | -0.24 |  | 3 | 252.2 | 43.92 | 0.000 |
| 1 | -0.07 |  |  | 2 | 253.4 | 45.09 | 0.000 |

**Table S6.** Summary of results from the piecewise SEM assessing relationships among male scent mark composition, socio-spatial behaviour, and relative fitness. Unstandardized coefficients (β), standard errors (SE), standardized coefficients (Std. β), and marginal pseudo-R^2^ (R^2^m) coefficients are provided. Bolded variables were retained in the best-ranked model (AICc = 1010.656). Coefficients for non-included variables are shown at their point of removal. RRS = relative reproductive success, RMS = relative mating success, SCI = sperm competition intensity, HQr = residency at high-quality site, CP/F = co-perchings per female guarded, DRI = dominance rank index, HL res = residuals of head length against SVL.

| **Response** | **Predictor** | ***β*** | **SE** | **Std. *β*** | ***F*** | **Df** | ***P*-value** | ***R^2^m*** |
| --- | --- | --- | --- | --- | --- | --- | --- | --- |
| ***RRS*** | **RMS** | **0.34** | **0.10** | **0.51** | **3.42** | **53** | **<0.001** |  |
|  | **SCI** | **-0.27** | **0.16** | **-0.38** | **-1.63** | **53** | **0.103** | **0.15** |
|  | **HQr** | **0.44** | **0.26** | **0.28** | **1.67** | **53** | **0.094** |  |
|  | CP/F | 0.09 | 0.09 | 0.17 | 0.98 | 53 | 0.325 |  |
|  | DRI | 0.07 | 0.15 | 0.10 | 0.47 | 53 | 0.641 |  |
| ***RMS*** | **Ergosterol** | **-0.42** | **0.13** | **-0.37** | **-3.26** | **78** | **0.001** |  |
|  | DRI | -0.03 | 0.13 | -0.03 | -0.24 | 78 | 0.810 |  |
|  | CP/F | 0.02 | 0.09 | 0.03 | 0.27 | 78 | 0.787 | 0.10 |
|  | HQr | 0.05 | 0.32 | 0.02 | 0.16 | 78 | 0.872 |  |
| ***SCI*** | **HQr** | **-0.71** | **0.31** | **-0.44** | **-2.28** | **53** | **0.022** |  |
|  | CP/F | -0.00 | 0.12 | -0.01 | -0.04 | 53 | 0.971 | 0.10 |
|  | DRI | -0.00 | 0.12 | -0.00 | -0.00 | 53 | 0.998 |  |
| ***DRI*** | **Ergosterol** | **-0.32** | **0.11** | **-0.32** | **-2.97** | **76** | **0.004** |  |
|  | α-Tocopherol | 0.19 | 0.11 | 0.19 | 1.68 | 75 | 0.097 |  |
|  | Cholesta-5,7-dien-3-ol | 0.25 | 0.13 | 0.25 | 1.97 | 74 | 0.052 |  |
|  | Oleic acid | -0.18 | 0.11 | -0.18 | -1.61 | 73 | 0.112 |  |
|  | Campesterol | 0.12 | 0.12 | 0.12 | 1.10 | 72 | 0.276 | 0.10 |
|  | Octadecanoic acid | -0.11 | 0.12 | 0.11 | -0.93 | 71 | 0.356 |  |
|  | Mass | 0.09 | 0.12 | 0.10 | 0.79 | 70 | 0.431 |  |
|  | HL res | 0.04 | 0.12 | 0.04 | 0.34 | 69 | 0.736 |  |
|  | Sitosterol | 0.02 | 0.12 | 0.02 | 0.16 | 68 | 0.870 |  |
| ***HQr*** | **DRI** | **1.68** | **0.22** | **0.60** | **3.64** | **78** | **<0.001** |  |
|  | **Campesterol** | **1.40** | **0.34** | **0.50** | **1.97** | **78** | **0.0483** |  |
|  | α-Tocopherol | -0.59 | 0.38 | -0.21 | -1.55 | 78 | 0.122 |  |
|  | Sitosterol | 0.37 | 0.51 | 0.12 | 0.73 | 78 | 0.463 | 0.58 |
|  | Oleic acid | -0.29 | 0.40 | -0.10 | -0.73 | 78 | 0.466 |  |
|  | HL res | -0.28 | 0.39 | -0.09 | -0.72 | 78 | 0.474 |  |
|  | Cholesta-5,7-dien-3-ol | -0.21 | 0.44 | -0.07 | -0.48 | 78 | 0.634 |  |
|  | Mass | 0.20 | 0.42 | 0.06 | 0.46 | 78 | 0.642 |  |
|  | Octadecanoic acid | -0.17 | 0.46 | -0.06 | -0.38 | 78 | 0.705 |  |
|  | Ergosterol | -0.07 | 0.70 | -0.02 | -0.10 | 78 | 0.917 |  |
| ***CP/F*** | **DRI** | **0.30** | **0.06** | **0.38** | **2.60** | **78** | **0.010** |  |
|  | **HQr** | **0.59** | **0.11** | **0.32** | **2.57** | **78** | **0.010** |  |
|  | **Oleic acid** | **0.21** | **0.05** | **0.26** | **2.16** | **78** | **0.031** |  |
|  | α-Tocopherol | -0.09 | 0.11 | -0.12 | -0.87 | 78 | 0.383 |  |
|  | Cholesta-5,7-dien-3-ol | -0.06 | 0.10 | -0.08 | -0.64 | 78 | 0.521 | 0.18 |
|  | Sitosterol | 0.06 | 0.11 | 0.08 | 0.54 | 78 | 0.590 |  |
|  | Campesterol | -0.08 | 0.12 | 0.08 | -0.64 | 78 | 0.520 |  |
|  | HL res | 0.03 | 0.11 | 0.04 | 0.257 | 78 | 0.798 |  |
|  | Mass | 0.01 | 0.11 | 0.01 | 0.05 | 78 | 0.964 |  |
|  | Ergosterol | -0.00 | 0.14 | -0.00 | -0.01 | 78 | 0.992 |  |
|  | Octadecanoic acid | -0.00 | 0.11 | -0.00 | -0.01 | 78 | 0.993 |  |

**Table S7.** Summary of model-averaged coefficients from the individual GLMMs nested as component models in the piecewise SEM. Variance inflation factors (VIF) correspond to the saturated model. Standardized coefficients (Std. *β*) represent the conditionally-averaged effect sizes across the subset of top models (ΔAICc < 4), with CI_95_, *Z*-values, and *P*-values. Relative importance values (RIV) represent the summed Akaike weights across all top models in which each predictor was retained, reflecting their relative support in explaining variation. Bolded letters highlight variables with RIV > 0.55. which are considered strong predictors. RRS = relative reproductive success, RMS = relative mating success, HQr = residency at HQ site, CP/F = co-perchings per female guarded, DRI = dominance rank index, HL res = residuals of head length against SVL.

| **Respose** | **Predictor** | ***N*** | **VIF** | **Std. *β*** | **CI_95_** | ***Z*** | ***P*-value** | **RIV** |
| --- | --- | --- | --- | --- | --- | --- | --- | --- |
| ***RRS*** | **RMS** |  | **1.19** | **0.38** | **[0.16, 0.60]** | **3.37** | **<0.001** | **1.00** |
|  | **SCI** |  | **1.47** | **-0.30** | **[-0.62, 0.01]** | **1.87** | **0.061** | **0.64** |
|  | HQr | 14 | 2.21 | 0.50 | [-0.10, 1.10] | 1.61 | 0.103 | 0.50 |
|  | CP/F |  | 1.51 | 0.16 | [-0.07, 0.38 | 1.35 | 0.178 | 0.39 |
|  | DRI |  | 1.62 | 0.14 | [-0.16, 0.44] | 0.93 | 0.354 | 0.30 |
| ***RMS*** | **Ergosterol** |  | **1.13** | **-0.05** | **[-0.67, -0.16]** | **3.17** | **0.002** | **1.00** |
|  | CP/F | 4 | 1.56 | 0.03 | [-0.19, 0.24] | 0.27 | 0.791 | 0.17 |
|  | HQr |  | 1.52 | 0.06 | [-0.46, 0.57] | 0.21 | 0.831 | 0.17 |
|  | DRI |  | 1.53 | -0.01 | [-0.25, 0.23] | 0.09 | 0.927 | 0.16 |
| ***SCI*** | **HQr** |  | **1.45** | **-0.71** | **[-1.39, -0.04]** | **2.07** | **0.038** | **0.84** |
|  | CP/F | 5 | 1.43 | -0.04 | [-0.28, 0.19] | 0.35 | 0.725 | 0.23 |
|  | DRI |  | 1.25 | -0.00 | [-0.24, 0.24] | 0.01 | 0.992 | 0.16 |
| ***DRI*** | **Ergosterol** |  | **1.75** | **-0.92** | **[-1.62, -0.22]** | **2.59** | **0.010** | **0.98** |
|  | **α-Tocopherol** |  | **1.49** | **0.65** | **[0.01, 1.30]** | **1.99** | **0.047** | **0.67** |
|  | **Cholesta-5,7-dien-3-ol** |  | **1.75** | **0.60** | **[-0.07, 1.28]** | **1.74** | **0.081** | **0.55** |
|  | Oleic acid | 40 | 1.33 | -0.49 | [-1.11, 0.13] | 1.54 | 0.124 | 0.36 |
|  | Mass |  | 1.27 | 0.35 | [-0.27, 0.98] | 1.12 | 0.265 | 0.20 |
|  | Campesterol |  | 1.53 | 0.32 | [-0.30, 0.94] | 1.01 | 0.311 | 0.19 |
|  | HL res |  | 1.11 | 0.18 | [-0.42, 0.78] | 0.59 | 0.555 | 0.10 |
|  | Octadecanoic acid |  | 1.39 | -0.17 | [-0.78, 0.44] | 0.56 | 0.579 | 0.09 |
|  | Sitosterol |  | 1.31 | 0.03 | [-0.54, 0.59] | 0.09 | 0.926 | 0.06 |
| ***HQr*** | **DRI** |  | **2.05** | **1.81** | **[0.77, 2.84]** | **3.43** | **0.001** | **1.00** |
|  | **Campesterol** |  | **3.27** | **1.33** | **[-0.08, 2.73]** | **1.85** | **0.064** | **1.00** |
|  | α-Tocopherol | 33 | 2.29 | -0.57 | [-1.37, 0.23] | 1.4 | 0.161 | 0.43 |
|  | Oleic acid |  | 1.42 | -0.42 | [-1.21, 0.38] | 1.03 | 0.302 | 0.28 |
|  | HL res |  | 1.19 | -0.35 | [-1.08, 0.38] | 0.93 | 0.352 | 0.28 |
|  | Sitosterol |  | 1.46 | 0.34 | [-0.59, 1.27] | 0.71 | 0.476 | 0.17 |
|  | Mass |  | 1.89 | 0.08 | [-0.68, 0.84] | 0.2 | 0.839 | 0.11 |
|  | Octadecanoic acid |  | 1.27 | -0.03 | [-0.75, 0.70] | 0.08 | 0.94 | 0.11 |
|  | Cholesta-5,7-dien-3-ol |  | 1.58 | -0.1 | [-0.85, 0.66] | 0.25 | 0.804 | 0.10 |
|  | Ergosterol |  | 3.60 | -0.07 | [-1.03, 0.88] | 0.15 | 0.883 | 0.09 |
| ***CP/F*** | **HQr** |  | **1.62** | **0.57** | **[0.11, 1.03]** | **2.42** | **0.016** | **1.00** |
|  | **DRI** |  | **1.8** | **0.31** | **[0.07, 0.54]** | **2.55** | **0.011** | **1.00** |
|  | **Oleic acid** |  | **1.4** | **0.21** | **[0.01, 0.40]** | **2.11** | **0.035** | **0.87** |
|  | α-Tocopherol | 16 | 1.74 | -0.09 | [-0.31, 0.12] | 0.86 | 0.388 | 0.20 |
|  | Sitosterol |  | 1.48 | 0.07 | [-0.15, 0.30] | 0.66 | 0.511 | 0.14 |
|  | Ergosterol |  | 2.32 | -0.05 | [-0.26, 0.15] | 0.53 | 0.593 | 0.10 |
|  | Cholesta-5,7-dien-3-ol |  | 1.91 | -0.05 | [-0.25, 0.15] | 0.51 | 0.612 | 0.10 |
|  | Mass |  | 1.63 | -0.05 | [-0.24, 0.14] | 0.54 | 0.588 | 0.09 |
|  | Campesterol |  | 1.85 | -0.05 | [-0.27, 0.17] | 0.44 | 0.658 | 0.09 |
|  | Octadecanoic acid |  | 1.4 | 0.01 | [-0.19, 0.20] | 0.05 | 0.958 | 0.06 |
|  | HL res |  | 1.18 | 0 | [-0.22, 0.21] | 0.04 | 0.966 | 0.06 |

**Table S8.** Standardized path coefficients (Std. *β*) and 95% confidence intervals (CI_95_) for direct and indirect effects estimated using bootstrapping (1,000 replicates). The table reports relationships among scent-mark composition and socio-spatial behaviour acting as mediators of male relative reproductive success as identified in the best-fitting piecewise SEM. Indirect effects are aggregated across all mediating pathways.

| **Response** | **Predictor** | ***Std. β*** | **Lower CI_95_** | **Upper CI_95_** | **Effect type** |
| --- | --- | --- | --- | --- | --- |
| *Reproductive success* | Mating success | 0.066 | 0.038 | 0.125 | Direct |
|  | Sperm competition intensity | -0.048 | -0.130 | -0.004 | Direct |
|  | High-quality residency | 0.038 | 0.002 | 0.094 | Direct |
|  | Ergosterol | -0.005 | -0.009 | -0003 | Indirect |
|  | Campesterol | 0.003 | 0.001 | 0.005 | Indirect |
|  | Dominance | 0.004 | 0.001 | 0.007 | Indirect |
|  | High-quality residency | 0.004 | -0.019 | 0.013 | Indirect |
| *Mating success* | Ergosterol | -0.051 | -0.069 | -0.030 | Direct |
| *Sperm competition intensity* | High-quality residency | -0.079 | -0.213 | -0.034 | Direct |
|  | Ergosterol | 0.002 | 0.001 | 0.008 | Indirect |
|  | Campesterol | -0.006 | -0.021 | -0.002 | Indirect |
|  | Dominance | -0.007 | -0.017 | -0.003 | Indirect |
| *Dominance* | Ergosterol | -0.032 | -0.043 | -0.017 | Direct |
| *High-quality residency* | Dominance | 0.089 | 0.056 | 0.143 | Direct |
|  | Campesterol | 0.074 | 0.035 | 0.147 | Direct |
|  | Ergosterol | -0.029 | -0.038 | -0.017 | Indirect |
| *Coperchings per female* | Dominance | 0.037 | 0.015 | 0.056 | Direct |
|  | High-quality residency | 0.031 | 0.019 | 0.046 | Direct |
|  | Oleic acid | 0.029 | 0.015 | 0.043 | Direct |
|  | Ergosterol | -0.013 | -0.023 | -0.005 | Indirect |
|  | Campesterol | 0.002 | 0.001 | 0.004 | Indirect |
|  | High-quality residency | 0.003 | 0.002 | 0.004 | Indirect |

**Table S9.** Summary of results from the piecewise SEM assessing relationships among male scent mark composition, socio-spatial behaviour, and relative fitness (excluding males without offspring). Unstandardized coefficients (β), standard errors (SE), standardized coefficients (Std. β), and marginal pseudo-R^2^ (R^2^m) coefficients are provided. Bolded variables were retained in the best-ranked model (AICc = 654.069). Coefficients for non-included variables are shown at their point of removal. RRS = relative reproductive success, RMS = relative mating success, HQr = residency at HQ site, CP/F = co-perchings per female guarded, DRI = dominance rank index, HL res = residuals of head length against SVL.

| **Respose** | **Predictor** | ***β*** | **SE** | **Std. *β*** | ***F*** | **Df** | ***P*-value** | ***R^2^m*** |
| --- | --- | --- | --- | --- | --- | --- | --- | --- |
| ***RRS*** | **RMS** | **0.34** | **0.10** | **0.25** | **3.49** | **53** | **<0.001** |  |
|  | **SCI** | **-0.27** | **0.16** | **-0.21** | **-1.68** | **53** | **0.093** | **0.23** |
|  | **HQr** | **0.44** | **0.26** | **0.16** | **1.71** | **53** | **0.089** |  |
|  | CP/F | 0.09 | 0.09 | 0.10 | 0.98 | 53 | 0.325 |  |
|  | DRI | 0.07 | 0.15 | 0.06 | 0.47 | 53 | 0.641 |  |
| ***RMS*** | Ergosterol | -0.11 | 0.13 | -0.11 | -0.86 | 53 | 0.392 |  |
|  | DRI | -0.10 | -0.10 | -0.11 | -0.72 | 53 | 0.471 |  |
|  | CP/F | -0.04 | 0.11 | -0.06 | -0.36 | 53 | 0.715 | 0.00 |
|  | HQr | 0.10 | 0.26 | 0.05 | 0.40 | 53 | 0.692 |  |
| ***SCI*** | **HQr** | **-0.71** | **0.31** | **-0.32** | **-2.28** | **53** | **0.022** |  |
|  | CP/F | -0.00 | 0.11 | -0.00 | 0.03 | 53 | 0.972 | 0.10 |
|  | DRI | -0.00 | 0.12 | -0.00 | -0.00 | 53 | 0.998 |  |
| ***DRI*** | **Ergosterol** | **-0.39** | **0.14** | -0.35 | **-2.74** | **50** | **0.008** |  |
|  | **Mass** | **0.26** | **0.13** | **0.26** | **2.01** | **50** | **0.050** |  |
|  | Cholesta-5,7-dien-3-ol | 0.21 | 0.15 | 0.22 | 1.42 | 49 | 0.162 |  |
|  | Sitosterol | 0.15 | 0.14 | 0.14 | 1.05 | 48 | 0.299 |  |
|  | Octadecanoic acid | 0.08 | 0.15 | 0.09 | 0.55 | 47 | 0.586 | 0.17 |
|  | HL res | 0.07 | 0.15 | 0.06 | 0.44 | 46 | 0.661 |  |
|  | Campesterol | 0.08 | 0.20 | 0.07 | 0.39 | 45 | 0.700 |  |
|  | α-Tocopherol | -0.02 | 0.16 | -0.02 | 1.68 | 44 | 0.097 |  |
|  | Oleic acid | 0.01 | 0.17 | 0.01 | 0.06 | 43 | 0.953 |  |
| ***HQr*** | **DRI** | **2.28** | **0.74** | **0.74** | **3.09** | **53** | **0.002** |  |
|  | **Campesterol** | **1.51** | **0.98** | **0.45** | **1.53** | **53** | **0.125** |  |
|  | Ergosterol | -1.50 | 1.11 | -0.32 | -1.34 | 53 | 0.181 |  |
|  | Sitosterol | 1.39 | 1.30 | 0.29 | 1.07 | 53 | 0.283 |  |
|  | Mass | 0.50 | 0.51 | 0.15 | 0.98 | 53 | 0.327 |  |
|  | α-Tocopherol | -0.42 | 0.47 | -0.13 | -0.90 | 53 | 0.370 | 0.64 |
|  | HL res | 0.24 | 0.56 | 0.05 | 0.43 | 53 | 0.670 |  |
|  | Octadecanoic acid | -0.20 | 0.63 | -0.04 | 0.32 | 53 | 0.750 |  |
|  | Cholesta-5,7-dien-3-ol | -0.11 | 0.79 | -0.02 | -0.13 | 53 | 0.895 |  |
|  | Oleic acid | -0.13 | 0.73 | -0.03 | -0.18 | 53 | 0.861 |  |
| ***CP/F*** | **HQr** | **0.96** | **0.23** | **0.32** | **4.26** | **53** | **<0.001** |  |
|  | **DRI** | **0.20** | **0.14** | **0.16** | **1.38** | **53** | **0.166** |  |
|  | **Oleic acid** | **0.19** | **0.11** | **0.15** | **1.71** | **53** | **0.087** |  |
|  | Campesterol | -0.15 | 0.19 | -0.11 | -0.79 | 53 | 0.431 |  |
|  | Ergosterol | 0.14 | 0.17 | 0.10 | 0.83 | 53 | 0.408 | 0.17 |
|  | HL res | 0.12 | 0.13 | 0.09 | 0.91 | 53 | 0.362 |  |
|  | Sitosterol | 0.10 | 0.18 | 0.08 | 0.54 | 53 | 0.586 |  |
|  | Octadecanoic acid | 0.09 | 0.13 | 0.07 | 0.69 | 53 | 0.489 |  |
|  | Cholesta-5,7-dien-3-ol | -0.07 | 0.12 | -0.06 | 0.54 | 53 | 0.584 |  |
|  | Mass | -0.06 | 0.14 | -0.05 | -0.42 | 53 | 0.674 |  |
|  | α-Tocopherol | 0.04 | 0.17 | 0.03 | 0.27 | 53 | 0.787 |  |

**Table S10.** Summary of model-averaged coefficients from the individual GLMMs nested as component models in the piecewise SEM (excluding males without offspring). Variance inflation factors (VIF) correspond to the saturated model. Standardized coefficients (Std. *β*) represent the conditionally-averaged effect sizes across the subset of top models (ΔAICc < 4), with CI_95_, *Z*-values, and *P*-values. Relative importance values (RIV) represent the summed Akaike weights across all top models in which each predictor was retained, reflecting their relative support in explaining variation. Bolded letters highlight variables with RIV > 0.55, which are considered strong predictors. RRS = relative reproductive success, RMS = relative mating success, HQr = residency at HQ site, CP/F = co-perchings per female guarded, DRI = dominance rank index, HL res = residuals of head length against SVL.

| **Respose** | **Predictor** | ***N*** | **VIF** | **Std. *β*** | **CI_95_** | ***Z*** | ***P*-value** | **RIV** |
| --- | --- | --- | --- | --- | --- | --- | --- | --- |
| ***RRS*** | **RMS** |  | **1.19** | **0.38** | **[0.16, 0.60]** | **3.37** | **<0.001** | **1.00** |
|  | **SCI** |  | **1.47** | **-0.30** | **[-0.62, 0.01]** | **1.87** | **0.061** | **0.64** |
|  | HQr | 14 | 1.52 | 0.50 | [-0.10, 1.10] | 1.61 | 0.103 | 0.50 |
|  | CP/F |  | 1.56 | 0.16 | [-0.07, 0.38 | 1.35 | 0.178 | 0.39 |
|  | DRI |  | 1.53 | 0.14 | [-0.16, 0.44] | 0.93 | 0.354 | 0.30 |
| ***RMS*** | Ergosterol |  | 1.14 | -0.12 | [-0.38, 0.15] | 0.85 | 0.398 | 0.27 |
|  | HQr |  | 1.72 | 0.11 | [-0.41, 0.63] | 0.42 | 0.674 | 0.18 |
|  | DRI | 7 | 1.65 | -0.02 | [-0.26, 0.21] | 0.19 | 0.849 | 0.17 |
|  | CP/F |  | 1.58 | -0.02 | [-0.24, 0.21] | 0.14 | 0.891 | 0.12 |
| ***SCI*** | **HQr** |  | **1.45** | **-0.71** | **[-1.39, -0.04]** | **2.07** | **0.038** | **0.84** |
|  | CP/F | 5 | 1.43 | -0.04 | [-0.28, 0.19] | 0.35 | 0.725 | 0.23 |
|  | DRI |  | 1.25 | -0.00 | [-0.24, 0.24] | 0.01 | 0.992 | 0.16 |
| ***DRI*** | **Ergosterol** |  | **2.60** | **-1.20** | **[-2.12, -0.27]** | **2.53** | **0.014** | **0.98** |
|  | **Mass** |  | **1.42** | **0.72** | **[0.01, 1.42]** | **2.00** | **0.046** | **0.69** |
|  | Cholesta-5,7-dien-3-ol |  | 2.35 | 0.57 | [-0.26, 1.39] | 1.35 | 0.178 | 0.38 |
|  | Octadecanoic acid | 32 | 1.74 | 0.41 | [-0.32, 1.13] | 1.11 | 0.269 | 0.23 |
|  | Campesterol |  | 1.99 | 0.18 | [-0.74, 1.11] | 0.39 | 0.700 | 0.13 |
|  | Sitosterol |  | 1.49 | 0.31 | [-0.46, 1.07] | 0.79 | 0.432 | 0.12 |
|  | HL res |  | 1.26 | 0.13 | [-0.63, 0.88] | 0.33 | 0.743 | 0.10 |
|  | Oleic acid |  | 1.62 | -0.11 | [-0.87, 0.66] | 0.27 | 0.786 | 0.10 |
|  | α-Tocopherol |  | 1.71 | -0.00 | [-0.79, 0.79] | 0.01 | 0.995 | 0.10 |
| ***HQr*** | **DRI** |  | **2.23** | **2.31** | **[0.72, 3.90]** | **2.85** | **0.004** | **1.00** |
|  | **Campesterol** |  | **5.37** | **1.53** | **[-0.86, 3.92]** | **1.26** | **0.209** | **0.67** |
|  | Sitosterol |  | 2.69 | 1.29 | [-0.81, 3.38] | 1.20 | 0.229 | 0.50 |
|  | Mass |  | 2.22 | 0.65 | [-0.61, 1.90] | 1.01 | 0.311 | 0.24 |
|  | Oleic acid |  | 2.25 | -0.45 | [-1.42, 0.53] | 0.90 | 0.370 | 0.19 |
|  | α-Tocopherol | 44 | 3.38 | -0.44 | [-1.51, 0.63] | 0.80 | 0.422 | 0.17 |
|  | Ergosterol |  | 5.76 | -0.37 | [-2.17, 1.43] | 0.41 | 0.685 | 0.14 |
|  | Octadecanoic acid |  | 2.08 | 0.28 | [-0.69, 1.26] | 0.57 | 0.568 | 0.11 |
|  | Cholesta-5,7-dien-3-ol |  | 2.30 | -0.08 | [-1.16, 1.01] | 0.14 | 0.892 | 0.08 |
|  | HL res |  | 1.47 | -0.18 | [-1.08, 0.72] | 0.39 | 0.698 | 0.06 |
| ***CP/F*** | **HQr** |  | **1.71** | **0.85** | **[0.30, 1.40]** | **3.03** | **0.002** | **1.00** |
|  | Oleic acid |  | 1.72 | 0.19 | [-0.04, 0.42] | 1.60 | 0.110 | 0.48 |
|  | DRI |  | 1.70 | 0.21 | [-0.09, 0.50] | 1.39 | 0.165 | 0.42 |
|  | HL res |  | 1.27 | 0.13 | [-0.13, 0.40] | 1.00 | 0.320 | 0.17 |
|  | Cholesta-5,7-dien-3-ol |  | 2.39 | -0.07 | [-0.31, 0.17] | 0.58 | 0.562 | 0.11 |
|  | Mass |  | 2.03 | -0.05 | [-0.30, 0.18] | 0.45 | 0.654 | 0.10 |
|  | α-Tocopherol | 40 | 2.04 | 0.03 | [-0.22, 0.28] | 0.25 | 0.803 | 0.09 |
|  | Sitosterol |  | 1.83 | 0.04 | [-0.24, 0.31] | 0.14 | 0.262 | 0.08 |
|  | Ergosterol |  | 2.94 | 0.03 | [-0.25, 0.30] | 0.19 | 0.853 | 0.08 |
|  | Campesterol |  | 2.41 | -0.03 | [-0.33, 0.26] | 0.23 | 0.822 | 0.08 |
|  | Octadecanoic acid |  | 1.76 | 0.01 | [-0.22, 0.24] | 0.11 | 0.916 | 0.08 |

**Table S11.** Standardized path coefficients (Std. *β*) and 95% confidence intervals (CI_95_) for direct and indirect effects estimated using bootstrapping (1,000 replicates). The table reports relationships among scent mark composition and socio-spatial behaviour acting as mediators of male relative reproductive success (RRS) as identified in the best-fitting piecewise SEM (excluding males without offspring). Indirect effects are aggregated across all mediating pathways.

| **Response** | **Predictor** | ***Std. β*** | **Lower CI_95_** | **Upper CI_95_** | **Effect type** |
| --- | --- | --- | --- | --- | --- |
| *Reproductive success* | Mating success | 0.067 | 0.040 | 0.130 | Direct |
|  | Sperm competition intensity | -0.049 | -0.130 | -0.004 | Direct |
|  | High-quality residency | 0.038 | 0.002 | 0.096 | Direct |
|  | Dominance | 0.005 | 0.002 | 0.014 | Indirect |
|  | High-quality residency | 0.004 | -0.025 | 0.014 | Indirect |
|  | Campesterol | 0.003 | 0.001 | 0.008 | Indirect |
|  | Ergosterol | -0.002 | -0.005 | -0.000 | Indirect |
|  | Mass | 0.001 | 0.000 | 0.004 | Indirect |
| *Sperm competition intensity* | High-quality residency | -0.079 | -0.213 | -0.034 | Direct |
|  | Dominance | -0.010 | -0.030 | -0.002 | Indirect |
|  | Campesterol | -0.006 | -0.032 | -0.001 | Indirect |
|  | Ergosterol | 0.003 | 0.000 | 0.022 | Indirect |
|  | Mass | -0.003 | -0.013 | 0.050 | Indirect |
| *Dominance* | Ergosterol | -0.035 | -0.056 | -0.014 | Direct |
|  | Mass | 0.026 | 0.002 | 0.046 | Direct |
| *High-quality residency* | Dominance | 0.124 | 0.074 | 0.275 | Direct |
|  | Campesterol | 0.075 | 0.024 | 0.225 | Direct |
|  | Ergosterol | -0.044 | -0.189 | -0.023 | Indirect |
|  | Mass | 0.032 | 0.008 | 0.123 | Indirect |
| *Coperchings per female* | Oleic acid | 0.029 | 0.08 | 0.049 | Direct |
|  | High-quality residency | 0.065 | 0.047 | 0.086 | Direct |
|  | Dominance | 0.008 | 0.004 | 0.018 | Indirect |
|  | Campesterol | 0.005 | 0.001 | 0.017 | Indirect |
|  | Ergosterol | -0.003 | -0.009 | -0.001 | Indirect |
|  | Mass | 0.002 | 0.001 | 0.009 | Indirect |

**Table S12.** Results from Bradley–Terry models examining the effects of morphology, chemical compounds, behavioural displays, prior contest history and residency on the log odds of winning a contest. M1 was fitted on the full dataset of contests. M2 was fitted on the early contests observed before Day 10. M3 included every late contest observed from day 10 onwards. Significant predictors are highlighted in bold (α = 0.95, *P-value* < 0.05). Statistics for non-significant factors are included at the point of their deletion from the model.

| **Model** | **Predicted** | **Variable** | **Estimate** | **SE** | **VIF** | ***Z*** | ***P-*value** |
| --- | --- | --- | --- | --- | --- | --- | --- |
| M1  All contests  544 fights | 83.8% | Mass | 0.24 | 0.23 | 1.47 | 1.04 | 0.296 |
|  |  | HL res | -0.11 | 0.22 | 1.27 | -0.50 | 0.620 |
|  |  | **Cholesta-5,7-dien-3-ol** | **0.59** | **0.24** | **2.17** | **2.47** | **0.013** |
|  |  | **Ergosterol** | **-0.61** | **0.28** | **2.35** | **-2.22** | **0.026** |
|  |  | Octadecanoic acid | -0.07 | 0.25 | 1.52 | -0.27 | 0.785 |
|  |  | Campesterol | 0.20 | 0.27 | 1.81 | 0.74 | 0.459 |
|  |  | Oleic acid | -0.14 | 0.23 | 1.41 | -0.64 | 0.523 |
|  |  | **α-Tocopherol** | **0.56** | **0.21** | **1.78** | **2.60** | **0.009** |
|  |  | Sitosterol | 0.30 | 0.24 | 1.59 | 1.24 | 0.213 |
|  |  | **Raised-body display** | **2.89** | **0.41** | **1.24** | **6.97** | **< 0.001** |
|  |  | Type II foot shakes | -1.17 | 0.67 | 1.06 | 1.74 | 0.082 |
|  |  | **Prior contest history** | **0.65** | **0.24** | **1.10** | **2.68** | **0.007** |
|  |  | **Resident** | **0.83** | **0.33** | **1.22** | **2.48** | **0.013** |
|  |  | ID (Std. dev) | 1.08 | 0.19 |  | 5.76 | <0.001 |
| M2  Early ≤ Day 9  137 fights | 71.5% | Mass | 0.32 | 0.28 | 1.39 | 1.15 | 0.250 |
|  |  | HL res | 0.09 | 0.31 | 1.50 | 0.31 | 0.760 |
|  |  | **Cholesta-5,7-dien-3-ol** | **0.76** | **0.30** | **2.86** | **2.52** | **0.012** |
|  |  | **Ergosterol** | **-1.30** | **0.44** | **2.58** | **-2.98** | **0.003** |
|  |  | Octadecanoic acid | -0.10 | 0.36 | 1.74 | -0.27 | 0.790 |
|  |  | Campesterol | 0.08 | 0.48 | 1.78 | 0.17 | 0.863 |
|  |  | Oleic acid | -0.09 | 0.34 | 1.61 | -0.28 | 0.784 |
|  |  | α-Tocopherol | 0.61 | 0.31 | 2.49 | 1.93 | 0.053 |
|  |  | Sitosterol | 0.07 | 0.32 | 1.52 | 0.23 | 0.815 |
|  |  | **Raised-body display** | **3.27** | **0.44** | **1.19** | **4.00** | **< 0.001** |
|  |  | Type II foot shakes | -0.86 | 1.22 | 1.24 | -0.70 | 0.481 |
|  |  | Prior contest history | -0.07 | 0.50 | 1.28 | -0.14 | 0.889 |
|  |  | Resident | 0.44 | 0.63 | 1.10 | 0.71 | 0.480 |
|  |  | ID (Std. dev) | 0.71 | 0.33 |  | 2.14 | 0.033 |
| M3  Late > Day 9  407 fights | 84.8% | Mass | 2.84 | 0.26 | 1.56 | 1.08 | 0.281 |
|  |  | HL res | -0.32 | 0.22 | 1.29 | -1.48 | 0.140 |
|  |  | Cholesta-5,7-dien-3-ol | 0.35 | 0.23 | 2.06 | 1.52 | 0.129 |
|  |  | Ergosterol | -0.44 | 0.30 | 2.19 | -1.47 | 0.143 |
|  |  | Octadecanoic acid | 0.01 | 0.28 | 1.51 | 0.03 | 0.980 |
|  |  | Campesterol | 0.03 | 0.33 | 1.91 | 0.10 | 0.922 |
|  |  | Oleic acid | 0.04 | 0.27 | 1.52 | -0.16 | 0.871 |
|  |  | **α-Tocopherol** | 0.48 | 0.22 | 1.83 | 2.22 | 0.026 |
|  |  | Sitosterol | 0.39 | 0.30 | 1.63 | 1.29 | 0.198 |
|  |  | **Raised-body display** | **2.79** | **0.50** | **1.28** | **5.53** | **< 0.001** |
|  |  | **Type II foot shakes** | **-2.29** | **1.12** | **1.17** | **-2.04** | **0.042** |
|  |  | **Prior contest history** | **1.02** | **0.30** | **1.14** | **3.43** | **< 0.001** |
|  |  | **Resident** | **0.82** | **0.41** | **1.28** | **2.01** | **0.044** |
|  |  | ID (Std. dev) | 1.08 | 0.22 |  | 4.92 | < 0.001 |

**Table S13.** Results of robust one-way ANOVAs (bootstrapped trimmed means) testing differences in ten chemical compounds among males settled in HQ sites, LQ sites, and the rocks in between. Each test reflects 10,000 bootstrap iterations. Reported are the mean ± SEM for each compound (as Z-scores) in each site quality category, test statistic, *P*-value, proportion of variance explained (% Var.), and effect size (ξ; ES).

| **Compound** | **HQ**  ***N =* 18** | **LQ**  ***N =* 24** | **Rocks**  ***N =* 36** | **Statistic** | ***P*-value** | **% Var.** | **ES** |
| --- | --- | --- | --- | --- | --- | --- | --- |
| Cholesta-5,7-dien-3-ol | 0.10 ± 0.27 | -0.18 ± 0.15 | 0.07 ± 0.18 | 0.31 | 0.732 | 0.021 | 0.146 |
| Ergosterol | -0.02 ± 0.27 | -0.18 ± 0.20 | 0.13 ± 0.16 | 0.91 | 0.409 | 0.030 | 0.172 |
| Hexadecanoic acid | 0.26 ± 0.16 | -0.10 ± 0.21 | -0.07 ± 0.19 | 0.29 | 0.776 | 0.047 | 0.216 |
| Octadecanoic acid | 0.11 ± 0.24 | -0.19 ± 0.20 | 0.07 ± 0.17 | 0.57 | 0.568 | 0.030 | 0.172 |
| Campesterol | 0.35 ± 0.13 | -0.15 ± 0.23 | -0.08 ± 0.18 | 0.31 | 0.745 | 0.044 | 0.209 |
| Ergosta-5,8-dien-3-ol | 0.13 ± 0.29 | -0.09 ± 0.18 | -0.00 ± 0.16 | 0.04 | 0.964 | 0.012 | 0.109 |
| Oleic acid | -0.24 ± 0.22 | -0.07 ± 0.20 | 0.17 ± 0.17 | 0.98 | 0.301 | 0.052 | 0.227 |
| α-Tocopherol | -0.08 ± 0.24 | 0.16 ± 0.20 | -0.07 ± 0.17 | 0.39 | 0.670 | 0.022 | 0.147 |
| Cholesterol | 0.07 ± 0.25 | 0.05 ± 0.21 | -0.07 ± 0.16 | 0.21 | 0.816 | 0.013 | 0.116 |
| Sitosterol | 0.24 ± 0.23 | -0.11 ± 0.20 | -0.05 ± 0.17 | 0.49 | 0.624 | 0.048 | 0.218 |

**Table S14.** Predictors of per-egg fertilisation success. Model-averaged coefficients for the beta-binomial models testing whether realised fertilisation success is explained by male-female spatial proximity, male scent-mark composition, or dominance. Variance inflation factors (VIF) correspond to the saturated model. Standardized coefficients (Std. *β*; log-odds per 1 SD) are conditionally-averaged across top models (ΔAICc < 4), with CI_95_, *Z*-values, and *P*-values. Relative importance (RIV) reflects summed Akaike weights across top models. Bold variables (RIV > 0.55; CI95 excluding zero) are considered strong predictors. “Same-site residency” is a binary indicator of whether male and female occupied the same pallet or stone. “Site-quality combination” is a four-level factor indicating the site-quality assigned to each of the possible female-male pair combinations. Missing lipid values (8 males) were imputed as 0 (mean z-score) to retain full choice sets; results excluding these males are in **Table S14**.

| **Model** | **Model including same-site residency** | | | | | | | |
| --- | --- | --- | --- | --- | --- | --- | --- | --- |
| **Response** | **Predictors** | **VIF** | **Std. *β*** | **CI_95_** | ***Z*** | ***P*-value** | ***N*** | **RIV** |
| *Fertilisation success*  *(per-egg)* | **Same-site residency** | **1.03** | **1.02** | **[0.41, 1.62]** | **3.27** | **0.001** | **37** | **1.00** |
|  | **Ergosterol** | **1.83** | −**0.55** | **[**−**0.85,** −**0.26]** | **3.71** | **<0.001** | **37** | **1.00** |
|  | Oleic acid | 1.16 | −0.19 | [−0.45, 0.06] | 1.47 | 0.142 | 19 | 0.49 |
|  | α-Tocopherol | 1.22 | 0.10 | [−0.16, 0.35] | 0.73 | 0.463 | 12 | 0.26 |
|  | Sitosterol | 1.26 | 0.12 | [−0.16, 0.41] | 0.84 | 0.401 | 10 | 0.24 |
|  | Cholesta-5,7-dien-3-ol | 1.52 | 0.14 | [−0.16, 0.45] | 0.91 | 0.364 | 10 | 0.22 |
|  | Dominance | 1.16 | 0.03 | [−0.06, 0.12] | 0.65 | 0.513 | 10 | 0.21 |
|  | Octadecanoic acid | 1.32 | 0.08 | [−0.17, 0.32] | 0.63 | 0.531 | 10 | 0.19 |
|  | Campesterol | 1.56 | 0.06 | [−0.21, 0.33] | 0.43 | 0.668 | 10 | 0.18 |
| **Model** | **Model including parental site-quality combination** | | | | | | | |
| **Response** | **Predictors** | **VIF** | **Std. *β*** | **CI_95_** | ***Statistic*** | ***P*-value** | ***N*** | **RIV** |
| *Fertilisation success*  *(per-egg)* | **Site-quality combination** | **1.30** | **-** | **-** | **21.20** | **<0.001** | **34** | **1.00** |
|  | **Ergosterol** | **1.90** | **–0.57** | **[–0.87, –0.27]** | **3.74** | **<0.001** | **34** | **1.00** |
|  | Oleic acid | 1.19 | –0.21 | [–0.47, 0.05] | 1.60 | 0.109 | 22 | 0.64 |
|  | Cholesta-5,7-dien-3-ol | 1.58 | 0.15 | [–0.16, 0.46] | 0.97 | 0.333 | 13 | 0.31 |
|  | α-Tocopherol | 1.24 | 0.11 | [–0.16, 0.37] | 0.79 | 0.429 | 9 | 0.22 |
|  | Sitosterol | 1.28 | 0.09 | [–0.20, 0.39] | 0.63 | 0.529 | 9 | 0.20 |
|  | Dominance | 1.32 | 0.03 | [–0.07, 0.13] | 0.58 | 0.565 | 8 | 0.18 |
|  | Campesterol | 1.63 | 0.05 | [–0.23, 0.32] | 0.34 | 0.735 | 7 | 0.15 |
|  | Octadecanoic acid | 1.36 | 0.02 | [–0.23, 0.28] | 0.17 | 0.864 | 7 | 0.15 |

**Table S15.** Predictors of per-egg fertilisation success. Model-averaged coefficients for the beta-binomial models excluding eight reproducing males with unknown lipids. Variance inflation factors (VIF) correspond to the saturated model. Standardized coefficients (Std. *β*; log-odds per 1 SD) are conditionally-averaged across top models (ΔAICc < 4), with CI_95_, *Z*-values, and *P*-values. Relative importance (RIV) reflects summed Akaike weights across top models. Bold variables (RIV > 0.55; CI95 excluding zero) are considered strong predictors. “Same-site residency” is a binary indicator of whether male and female occupied the same pallet or stone. “Site-quality combination” is a four-level factor indicating the site-quality assigned to each of the possible female-male pair combinations. For results with the full set of males (imputing lacking lipid values as 0, the mean of the z-distribution) see **Table S13**.

|  | **Model** | **Model including same-site residency** | | | | | | | | | | | | | | | |
| --- | --- | --- | --- | --- | --- | --- | --- | --- | --- | --- | --- | --- | --- | --- | --- | --- | --- |
|  | **Response** | **Predictors** | | **VIF** | | **Std. *β*** | | **CI_95_** | | | ***Z*** | | ***P*-value** | | ***N*** | | **RIV** |
|  | *Fertlisation success*  *(per-egg)* | **Same-site residency** | | **1.04** | | **1.59** | | **[1.10, 2.09]** | | | **6.32** | | **<0.001** | | **44** | | **1.00** |
|  |  | **Ergosterol** | | **1.86** | | **–0.66** | | **[–1.02, –0.30]** | | | **3.61** | | **<0.001** | | **44** | | **1.00** |
|  |  | Oleic acid | | 1.17 | | –0.21 | | [–0.51, 0.09] | | | 1.37 | | 0.172 | | 21 | | 0.47 |
|  |  | Campesterol | | 1.56 | | 0.19 | | [–0.15, 0.53] | | | 1.08 | | 0.279 | | 13 | | 0.30 |
|  |  | Dominance Rank Index | | 1.21 | | 0.05 | | [–0.06, 0.17] | | | 0.87 | | 0.385 | | 13 | | 0.25 |
|  |  | Cholesta-5,7-dien-3-ol | | 1.55 | | 0.12 | | [–0.24, 0.48] | | | 0.66 | | 0.509 | | 12 | | 0.21 |
|  |  | Sitosterol | | 1.26 | | 0.11 | | [–0.20, 0.43] | | | 0.70 | | 0.486 | | 12 | | 0.22 |
|  |  | α-Tocopherol | | 1.24 | | 0.07 | | [–0.25, 0.40] | | | 0.44 | | 0.657 | | 12 | | 0.19 |
|  |  | Octadecanoic acid | | 1.32 | | 0.04 | | [–0.26, 0.33] | | | 0.24 | | 0.808 | | 11 | | 0.17 |
|  | **Model** | **Model including parental site-quality combination** | | | | | | | | | | | | | | | |
|  | **Response** | **Predictors** | | **VIF** | | **Std. *β*** | | **CI_95_** | | | ***Statistic*** | | ***P*-value** | | ***N*** | | **RIV** |
| *Fertlisation success*  *(per-egg)* | | | **Site-quality combination** | | **1.33** | | **-** | | **-** | **χ^2^ = 18.94** | | **<0.001** | | **31** | | **1.00** | |
|  |  |  | **Ergosterol** | | **1.94** | | **–0.57** | | **[–0.87, –0.27]** | ***Z =* 3.74** | | **0.00018** | | **31** | | **1.00** | |
|  |  |  | Oleic acid | | 1.20 | | –0.21 | | [–0.47, 0.05] | *Z =* 1.60 | | 0.109 | | 21 | | 0.66 | |
|  |  |  | Cholesta-5,7-dien-3-ol | | 1.61 | | 0.15 | | [–0.16, 0.46] | *Z =* 0.97 | | 0.334 | | 12 | | 0.31 | |
|  |  |  | α-Tocopherol | | 1.27 | | 0.11 | | [–0.16, 0.37] | *Z =* 0.79 | | 0.429 | | 9 | | 0.24 | |
|  |  |  | Sitosterol | | 1.28 | | 0.09 | | [–0.20, 0.39] | *Z =* 0.63 | | 0.529 | | 9 | | 0.21 | |
|  |  |  | Campesterol | | 1.63 | | 0.05 | | [–0.23, 0.33] | *Z =* 0.36 | | 0.716 | | 6 | | 0.13 | |
|  |  |  | Octadecanoic acid | | 1.37 | | 0.02 | | [–0.23, 0.28] | *Z =* 0.17 | | 0.867 | | 6 | | 0.14 | |
|  |  |  | Dominance Rank Index | | 1.39 | | 0.03 | | [–0.07, 0.13] | *Z =* 0.58 | | 0.562 | | 6 | | 0.16 | |

5. **Figures**

**Figure S1.** Scatterplots showing the relationship between the logarithm of corresponding concentration levels in the scan-mode (X axis) and targeted analysis (Y axis), for each of the nine repeatedly analysed compounds. Black dots represent readings for the same individual. Blank readings in the scan-mode analysis (in red) have been substituted with zeroes and hence form a column close to the X axis origin. The wide span of these red datapoints in the Y axis suggests that these blank readings do not necessarily correspond to low concentration levels, and hence argues against the common practice of substituting these blank readings from scan-mode analysis with zeroes. R^2^ values correspond to Pearson correlation coefficients between non-zero values from the scan mode analysis and corresponding individual readings from the targeted analysis (excluding the red datapoints).

**Figure S2.** Boxplots showing the distribution of the relative concentration levels (corrected by individual secretion volume) estimated for 10 selected lipidic compounds. All variables have been transformed to z-scores to enable meaningful comparison among variables with differing scales. A) Untransformed readings relative to standard show both positive skew and extreme high-value outliers. B) Winsorized data capped at the 95^th^ percentile. C) Increased normality in the distribution of datapoints after adaptive pseudo-count addition and logarithmic transformation of the winsorized data.

**Figure S3.** Morphological predictors of male fitness. Correlation plot showing the relationship between the lipidic compound levels examined, and the four morphological variables measured for each individual male. Numbers and gradient cell colour in the upper panel indicate Pearson correlation coefficients, with asterisks representing significant pairwise correlations at the 95% confidence level. Compounds included in the subset showing no pairwise Pearson coefficient higher than 0.6 are indicated in green. Scatter plots in the lower panel correspond to individual datapoints, with colour fill representing alternative throat colours.

**Figure S4.** Spearman correlation matrix between raw, untransformed compound abundances and key socio-spatial and reproductive variables. Dominance showed the broadest associations across compounds, whereas relative mating and reproductive success were also strongly and negatively associated with ergosterol. Overall, patterns in the raw data were consistent with the main analyses after data preprocessing, supporting the robustness of the identified relationships. Asterisks indicate statistical significance (*P* < 0.05, *P* < 0.01, *P* < 0.001).

**Figure S5.** Correlation plot showing the relationship between male dominance rank, socio-spatial behaviour, and fitness variables. Numbers and gradient cell colour in the upper panel indicate Spearman correlation coefficients, with asterisks representing significant pairwise correlations at the 95% confidence level after correction for multiple comparisons. Scatter plots in the lower panel correspond to individual datapoints, with colour fill representing residency status at high-quality sites, low-quality sites, or the rocks in between (also considered low-quality sites).

**Figure S6.** Network of causal pathways fitted in the initial piecewise structural equation modelling (pSEM) procedure, representing the directional hypotheses relating morphological predictors, scent mark composition, socio-spatial behaviour, and relative reproductive fitness in male *Podarcis muralis*. Black arrows indicate paths specified in the original model formula. The blue arrow was added based on significant results from tests of *d*-separation. Additionally, a second model excluding sperm competition intensity (SCI) was run to assess its influence on the network.
